# Disentangling Qualitatively Different Faking Strategies in High-Stakes Personality Assessments: A Mixture Extension of the Multidimensional Nominal Response Model

**DOI:** 10.1177/00131644251341843

**Published:** 2025-07-29

**Authors:** Timo Seitz, Ö. Emre C. Alagöz, Thorsten Meiser

**Affiliations:** 1 University of Mannheim, Mannheim, Germany

**Keywords:** faking, mixture model, response strategies, high-stakes assessments, multidimensional item response theory

## Abstract

High-stakes personality assessments are often compromised by faking, where test-takers distort their responses according to social desirability. Many previous models have accounted for faking by modeling an additional latent dimension that quantifies each test-taker’s degree of faking. Such models assume a homogeneous response strategy among all test-takers, reflected in a measurement model in which substantive traits and faking jointly influence item responses. However, such a model will be misspecified if, for some test-takers, item responding is only a function of substantive traits or only a function of faking. To address this limitation, we propose a mixture modeling extension of the multidimensional nominal response model (M-MNRM) that can be used to account for qualitatively different response strategies and to model relationships of strategy use with external variables. In a simulation study, the M-MNRM exhibited good parameter recovery and high classification accuracy across multiple conditions. Analyses of three empirical high-stakes datasets provided evidence for the consistent presence of the specified latent classes in different personnel selection contexts, emphasizing the importance of accounting for such kind of response behavior heterogeneity in high-stakes assessment data. We end the article with a discussion of the model’s utility for psychological measurement.

## Introduction

Self-report personality questionnaires are frequently employed in high-stakes assessments like personnel selection (Diekmann & König, 2015; Nikolaou & Foti, 2018), as personality measures derived from self-report questionnaires have been found to predict job performance and other outcomes in various contexts (e.g., Ones et al., 2007; Sackett & Walmsley, 2014). However, considering that personality tests in high-stakes assessments carry important consequences for test-takers, there is the threat that test-takers deliberately present themselves in an overly favorable manner, that is, engage in faking. Many studies over the past few decades have shown that faking has several adverse effects on the psychometric properties of a test (Ziegler et al., 2011), including elevated mean scores (e.g., Birkeland et al., 2006), inflated correlations between trait scales (e.g., Christiansen et al., 2021), and biased rank orders of test-takers which ultimately alter selection decisions (e.g., Mueller-Hanson et al., 2003).

To account for the response bias of faking, several latent variable models have been developed to capture variance in item responses that is due to faking (e.g., Hendy et al., 2021; Seitz et al., 2024, 2025; Ziegler et al., 2015). These models typically assume a homogeneous response strategy among test-takers, reflected in a measurement model with a continuous latent faking dimension on which test-takers vary quantitatively. However, research has shown that test-takers in high-stakes assessments in fact employ qualitatively different response strategies (Griffith & Converse, 2011; Robie et al., 2007). In this case, a single measurement model does not fully capture the faking process and potentially yields biased estimates of person and item parameters.

In the present work, we address this limitation by extending the faking model by Seitz et al. (2024, 2025) in a mixture modeling framework. Throughout this article, we investigate the extended model in a simulation study and in a set of empirical datasets from three job application settings. Before presenting details about the model extension, we will first introduce previous model-based faking accounts as well as previous mixture modeling approaches.

### Model-Based Approaches to Accounting for Faking

As mentioned above, there are multiple approaches that account for faking using latent variable modeling. One prominent approach is to use structural equation modeling and model responses from a personality inventory with a bifactor model (Hendy et al., 2021; see also Klehe et al., 2012; Schmit & Ryan, 1993). In such a model, all items load on a common general factor, which captures variance among the items of different trait scales and thus reflects an “ideal-employee” or faking factor. The items of the different trait scales additionally load on specific factors, which reflect the respective substantive personality traits after controlling for the general (faking) factor. Previous work fitting a bifactor model to personality data has found that substantive trait estimates based on scores of specific factors are less distorted by faking than trait estimates based on classical scale scores (Hendy et al., 2021), and that the general faking factor is related to external covariates (Klehe et al., 2012).

Another approach to accounting for faking is to apply multidimensional item response theory (IRT). Multidimensional IRT models have frequently been used to account for response styles in rating scale data by specifying additional latent dimensions that reflect the response styles of interest (see Henninger & Meiser, 2020, for an overview). In many cases, these models are applications of the multidimensional nominal response model (MNRM; Takane & de Leeuw, 1987). In the parameterization of the model by Falk and Cai (2016; see also Thissen & Cai, 2016), item responses are modeled through the following softmax function which accounts for the influence of D latent dimensions:

(1) $$p\left(Y_{\mathrm{ni}}=k\;|\;\alpha_{i},S_{i},\gamma_{i},\theta_{n}\right)=\frac{\exp({\left(\alpha_{i}{}^{\circ}s_{\mathrm{ik}}\right)}^{'}\theta_{n}+\gamma_{\mathrm{ik}})}{\sum_{m=0}^{K}\exp({\left(\alpha_{i}{}^{\circ}s_{\mathrm{im}}\right)}^{'}\theta_{n}+\gamma_{\mathrm{im}})}\;.$$

Y∈{0,1,…,k,…,K} is a random variable representing an item response, with k denoting its realization (i.e., the selected response category). The probability of response category k selected by person n on item i depends on a vector of person parameters $\theta_{n}$ (containing trait scores of person n on the D dimensions), a vector of item-category intercepts $\gamma_{i}$ (containing intercept values of item i for the K+1 categories), a vector of item slopes $\alpha_{i}$ (containing factor loadings [aka discrimination parameters] of item i with respect to the D dimensions), and a matrix of scoring weights $S_{i}$ . Scoring weights reflect the relationship between category k and dimension d on item i , and can hence be used to specify the latent dimensions that should be modeled. For modeling substantive traits, a scoring weight vector of evenly spaced integer values is typically specified, following the partial credit model (Masters, 1982) for ordinal responses as a special case of the nominal response model (Bock, 1972; Thissen & Steinberg, 1986). For response styles such as extreme response style (ERS) or midscale response style (MRS), a 0/1 scoring scheme is usually employed, where categories triggered by high levels of the respective response styles are coded as 1 and other categories are coded as 0.

Seitz et al. (2024, 2025) transferred the method of specifying scoring weights to the response bias of faking. In particular, they set scoring weights of a faking dimension to values representing the desirability of a response category on a given item. When modeling responses to a personality test designed to measure three substantive traits with a 7-point Likert scale, the scoring weight matrix $S_{i}$ for an item measuring the first substantive trait can be denoted as:

(2) $$S_{i}=\left(\begin{array}{ccccccc} 0 & 1 & 2 & 3 & 4 & 5 & 6\\ 0 & 0 & 0 & 0 & 0 & 0 & 0\\ 0 & 0 & 0 & 0 & 0 & 0 & 0\\ \mathrm{de}s_{i0} & \mathrm{de}s_{i1} & \mathrm{de}s_{i2} & \mathrm{de}s_{i3} & \mathrm{de}s_{i4} & \mathrm{de}s_{i5} & \mathrm{de}s_{i6} \end{array}\right)\;,$$

where scoring weights of the substantive trait dimensions not measured by the particular item are set to 0 and $\mathrm{de}s_{\mathrm{ik}}$ stands for category k ’s desirability on item i . To get the desirability values in an empirical setting, one can conduct a pilot study in which participants are instructed to rate the desirability of each category of each item with respect to the social context of the actual personality testing (typically with respect to an application for a particular job; Seitz et al., 2025). Usually, items vary in how desirable the respective categories are, and the relationship between categories and desirability is oftentimes not strictly monotonic (Kuncel & Tellegen, 2009). Thus, when specifying the faking dimension as described, item-specific and potentially nonmonotonic faking effects can be modeled. In previous work, this way of modeling faking has been shown to significantly improve model fit, debias inflated correlations between substantive traits (Seitz et al., 2025), and enhance the estimation of individual substantive trait scores (Seitz et al., 2024).

### Mixture Modeling Approaches to Accounting for Heterogeneity in Response Behavior

Independent of the modeling of faking, there are other methods to account for different kinds of heterogeneity in response behavior. One parametric approach is to use mixture modeling. These models assume that the data consists of distinct, unobserved subpopulations, so-called latent classes, each of which is associated with a separate measurement model (e.g., McLachlan & Peel, 2000; von Davier & Rost, 2006). That is, parameter values or the entire model structure may not be constant for all test-takers but vary between classes. Substantively, the different classes are assumed to represent distinct response processes, varying response strategies, or other types of heterogeneity. To account for such heterogeneous response behavior, separate measurement models are estimated based on assigning test-takers to the different classes. As a result, mixture models yield estimates of class proportions and probabilities of class membership for each test-taker.

In psychometrics, mixture models have been extensively used to account for heterogeneity in item responding. Many studies have followed an exploratory approach. For example, mixture-distribution IRT models have been applied to study heterogeneity in the use of rating scales (Rost, 1991). Such analyses have often found two classes characterized by varying threshold distances, typically treated as manifestations of ERS (e.g., Böckenholt & Meiser, 2017; Eid & Rauber, 2000; Gollwitzer et al., 2005). However, the interpretation of classes in exploratory mixture models is usually post hoc, atheoretical, and can become cumbersome in the case of complex model structures.

As opposed to fully exploratory mixture models, there are confirmatory mixture modeling approaches. These models impose theoretically motivated parameter constraints to account for specific forms of heterogeneity. A prominent example of such models are HYBRID models (Yamamoto, 1987, 1989), which assume two classes with model structures related to different cognitive processes. One class is specified in terms of a regular IRT model, whereas a model of stochastic independence is specified in the second class (see von Davier & Yamamoto, 2004; Yamamoto & Everson, 1995; for extensions). Thus, the HYBRID model separates regular test-takers from test-takers who choose response categories randomly. An overview of mixture-distribution IRT and HYBRID models can be found in von Davier and Yamamoto (2007).

Furthermore, other confirmatory mixture models have been developed in recent years to account for heterogeneity in rating scale responses. Tijmstra et al. (2018) proposed a two-class mixture IRT model that models a qualitatively different use of the midpoint category of a rating scale. Similar to a HYBRID model, a regular (in this case, ordinal) IRT model is specified in one class, whereas the second class is parameterized in terms of an item response tree (IRTree) model where test-takers first decide whether to choose the midpoint category and then, given they have not chosen the midpoint category, indicate their actual endorsement level. The model thus separates test-takers who use the midpoint category as part of the ordinal scale for item endorsement from test-takers who treat the midpoint category as sort of a non-response. Similarly, Kim and Bolt (2021) developed a two-class mixture model that accounts for differences in how test-takers come to select extreme response categories. In this model, one class is specified in which the selection of extreme categories is determined by ERS, whereas the substantive trait influences the choice of extreme categories in the second class. That is, the model allows for interindividual differences regarding which latent dimensions affect item responses. Finally, Alagöz and Meiser (2024) proposed a four-class model in which the mixture components reflect a different use of ERS and MRS. To specify the classes, slopes of response style dimensions that are not used in a class are fixed to 0, leading to an “ERS-only class,”“MRS-only class,”“ERS&MRS class,” and “no response style class.” The model hence aims to detect fine-grained heterogeneity in test-takers’ response style usage. In data applications of these three mixture models, all classes turned out to be empirically prevalent, providing evidence for the existence of heterogeneous response strategies in questionnaire data.

### Heterogeneity of Response Strategies in High-Stakes Assessments

As noted above, previous faking models typically account for faking in terms of a latent variable that captures each test-taker’s faking degree. That is, a single measurement model is specified in which item responding is a function of test-takers’ substantive trait *and* faking levels. However, as for response styles, there is evidence that test-takers in high-stakes assessments do not only differ quantitatively in faking but also qualitatively. Evidence for this claim comes, for instance, from studies examining the prevalence of faking. These studies have come to the conclusion that many test-takers in high-stakes assessments do engage in faking but also that a considerable proportion do not show self-presentational behavior (see Griffith & Converse, 2011; for an overview). Griffith et al. (2007), for example, retested job applicants under anonymous conditions and observed that 30% to 50% of applicants had significantly elevated their scores in the preceding application whereas the remaining applicants had not (see also Arthur et al., 2010). Similarly, using the randomized response technique, König et al. (2011) found that 32% of applicants in the United States exaggerate positive features in job application contexts whereas the other 68% do not (see also Donovan et al., 2003). Furthermore, evidence for qualitative differences in test-takers’ faking behavior is provided by studies investigating the thought process in high-stakes personality testings (Robie et al., 2007; Röhner et al., 2025). Robie et al. (2007), for example, asked test-takers to think aloud while they were responding to a personality questionnaire under high-stakes conditions. Based on an analysis of verbal protocols, they found three groups: a group of test-takers referring to themselves and the “ideal” applicant while responding, a group of test-takers only considering themselves, as well as a group of test-takers exclusively responding from the perspective of the “ideal” applicant.

Considering this line of research, it is questionable whether the response process associated with faking can be described by a single continuous faking variable and a homogeneous loading structure of substantive traits and faking across test-takers. If, for subsets of test-takers, item responding is only a function of substantive traits or, to the other extreme, only a function of faking, a model assuming a joint influence of substantive traits and faking for all test-takers will be misspecified. For test-takers only responding according to substantive traits, the model will be inappropriate because substantive trait scores will be adjusted for an estimated faking degree that has no actual foundation since faking has not influenced item responding. Likewise, for test-takers for whom item responding is only a function of faking, the model will be inappropriate because it will nonetheless yield substantive trait score estimates for these test-takers. Apart from an inappropriate estimation of person parameters, one can also expect that the estimation of item parameters and latent correlations will be biased if test-takers differ qualitatively in how they (do not) align responses with desirability (see the simulation below).

## Mixture Multidimensional Nominal Response Model (M-MNRM)

To allow for different response strategies in the modeling of faking, we propose a mixture extension of the MNRM that Seitz et al. (2024, 2025) used to account for faking. We hereby follow a confirmatory mixture modeling approach by constraining class-specific model parameters based on the definition of classes (see Alagöz & Meiser, 2024, for a similar approach). In particular, we specify three latent classes reflecting the response strategies test-takers may use in high-stakes assessments (see the above-described study by Robie et al., 2007). The first class represents a response strategy where test-takers select categories based on a combination of substantive traits and a faking dimension (“S&F class”), which is equivalent to the measurement model in the non-mixture version of the MNRM. Such a response behavior is conceivable because the obvious goal of making a favorable impression conflicts with the goal of staying true to oneself (Kuncel et al., 2011), so test-takers may want to find a compromise. Also, the conflict between wanting the job and social norms like telling the truth may lead to the joint consideration of substantive traits and faking. The second class reflects a response strategy where test-takers only respond based on substantive traits while faking does not influence item responses (“S-only class”). Reasons for such a response behavior can be that some test-takers do not know how to portray themselves favorably and hence do not engage in faking (Marcus, 2009; Ziegler, 2011), that some are afraid of being detected as liar or imposter (Röhner et al., 2025; Turner, 2022), or that some deliberately want to be honest in order to avoid being selected for a job they do not fit to (Kanfer et al., 2001; Saks & Ashforth, 2002). The third class represents a response strategy where item responding is not determined by substantive traits but solely by a faking dimension (“F-only class”). Such a response behavior can occur when test-takers want to be hired at any cost and therefore only consider desirability aspects of the items, or when test-takers want to compensate for poor scores on other relevant selection criteria (such as cognitive ability tests or grades).

To implement the three classes in the mixture model, one can impose class-specific model constraints, namely, set slopes of latent dimensions that are not part of a given response strategy to 0 for the respective class. Technically, the model equation of the mixture MNRM (M-MNRM) can be written as:

(3) $$p\left(Y_{\mathrm{ni}}=k\;|\;\alpha_{\mathrm{ic}},S_{i},\gamma_{\mathrm{ic}},\theta_{n}\right)=\sum_{c=1}^{3}\frac{\exp\left({\left(\alpha_{\mathrm{ic}}{}^{\circ}s_{\mathrm{ik}}\right)}^{'}\theta_{n}+\gamma_{\mathrm{ikc}}\right)}{\sum_{m=0}^{K}\exp\left({\left(\alpha_{\mathrm{ic}}{}^{\circ}s_{\mathrm{im}}\right)}^{'}\theta_{n}+\gamma_{\mathrm{imc}}\right)}p(\zeta_{n}=c)\;,$$

where $\zeta_{n}\in \{1,\;2,\;3\}$ denotes the class membership of person n . This equation describes the total probability of response k for person n on item i by multiplying the class-specific response probability with the probability of being a member of this class before summing across the three classes. The term $p(\zeta_{n}=c)$ is often referred to as the proportion of class c . When conditioning on person n ’s class membership, the equation boils down to the class-specific probability of response k for person n on item i :

(4) $$p\left(Y_{\mathrm{ni}}=k\;|\;\alpha_{\mathrm{ic}},S_{i},\gamma_{\mathrm{ic}},\theta_{n},\zeta_{n}=c\right)=\frac{\exp({\left(\alpha_{\mathrm{ic}}{}^{\circ}s_{\mathrm{ik}}\right)}^{'}\theta_{n}+\gamma_{\mathrm{ikc}})}{\sum_{m=0}^{K}\exp({\left(\alpha_{\mathrm{ic}}{}^{\circ}s_{\mathrm{im}}\right)}^{'}\theta_{n}+\gamma_{\mathrm{imc}})}\;.$$

Consider modeling faking in a personality questionnaire measuring three substantive traits with three items each, the slope matrices of the three latent classes in the M-MNRM can be denoted as:

(5) $$A_{c=1}=\left(\begin{array}{cccc} \alpha_{1S_{1}} & 0 & 0 & \alpha_{1F}\\ \alpha_{2S_{1}} & 0 & 0 & \alpha_{2F}\\ \alpha_{3S_{1}} & 0 & 0 & \alpha_{3F}\\ 0 & \alpha_{4S_{2}} & 0 & \alpha_{4F}\\ 0 & \alpha_{5S_{2}} & 0 & \alpha_{5F}\\ 0 & \alpha_{6S_{2}} & 0 & \alpha_{6F}\\ 0 & 0 & \alpha_{7S_{3}} & \alpha_{7F}\\ 0 & 0 & \alpha_{8S_{3}} & \alpha_{8F}\\ 0 & 0 & \alpha_{9S_{3}} & \alpha_{9F} \end{array}\right)\;{\mathrm{for}\;\mathrm{the}\;}^{\prime \prime }S\&F\;\mathrm{class}{,}^{\prime \prime }$$

(6) $$A_{c=2}=\left(\begin{array}{cccc} \alpha_{1S_{1}} & 0 & 0 & 0\\ \alpha_{2S_{1}} & 0 & 0 & 0\\ \alpha_{3S_{1}} & 0 & 0 & 0\\ 0 & \alpha_{4S_{2}} & 0 & 0\\ 0 & \alpha_{5S_{2}} & 0 & 0\\ 0 & \alpha_{6S_{2}} & 0 & 0\\ 0 & 0 & \alpha_{7S_{3}} & 0\\ 0 & 0 & \alpha_{8S_{3}} & 0\\ 0 & 0 & \alpha_{9S_{3}} & 0 \end{array}\right)\;{\mathrm{for}\;\mathrm{the}\;}^{“}S-\mathrm{only}\;\mathrm{class},”\;\mathrm{and}$$

(7) $$A_{c=3}=\left(\begin{array}{cccc} 0 & 0 & 0 & \alpha_{1F}\\ 0 & 0 & 0 & \alpha_{2F}\\ 0 & 0 & 0 & \alpha_{3F}\\ 0 & 0 & 0 & \alpha_{4F}\\ 0 & 0 & 0 & \alpha_{5F}\\ 0 & 0 & 0 & \alpha_{6F}\\ 0 & 0 & 0 & \alpha_{7F}\\ 0 & 0 & 0 & \alpha_{8F}\\ 0 & 0 & 0 & \alpha_{9F} \end{array}\right)\;{\mathrm{for}\;\mathrm{the}\;}^{“}F-\mathrm{only}\;\mathrm{class},”$$

where the rows reflect the items and the columns reflect the latent dimensions.

Furthermore, to model relationships between the use of response strategies and external variables, class membership can be predicted by a set of covariates. This can be achieved through a latent multinomial logistic regression of class membership on the covariates:

(8) $$\pi_{\mathrm{nc}}=p\left(\zeta_{n}=c\;|\;X_{n}=x_{n}\right)=\frac{\exp(\beta_{0c}+\sum_{p=1}^{P}\beta_{\mathrm{pc}}x_{\mathrm{np}})}{\sum_{m=1}^{3}\exp(\beta_{0m}+\sum_{p=1}^{P}\beta_{\mathrm{pm}}x_{\mathrm{np}})}\;.$$

X is a multivariate random variable representing the P covariates, $x_{n}$ denotes the realizations for person n . $\beta_{0c}$ is the regression intercept for class c , $\beta_{\mathrm{pc}}$ are regression slopes that reflect the effect of covariate p on class c . In this article, the “S&F class” represents the reference class. Hence, regression coefficients pertaining to this class are fixed to 0, such that intercepts and slopes pertaining to the other classes are to be interpreted with respect to the “S&F class.”

It is important to note that the model does *not* imply that test-takers in the “S-only class” per se do not have a faking person parameter or, correspondingly, that test-takers in the “F-only class” per se do not have substantive trait person parameters. Instead, the model assumes that test-takers in the “S-only class” (“F-only class”) do not consider the faking dimension (substantive trait dimensions) when responding to the items. Similarly, the proposed model is not equivalent to a non-mixture model in which “S-only” test-takers (“F-only” test-takers) have faking person parameters (substantive trait person parameters) of 0. Whereas a person parameter of 0 simply reflects one possible value on the dimension’s latent continuum (oftentimes representing the latent mean), an item slope of 0 implies that the dimension does not explain any variance in item responses, which captures the idea of qualitatively different response strategies (see also Alagöz & Meiser, 2024). More information on this matter, including a data illustration, can be found in the Supplemental Material.

Note also that, in our parameterization, non-fixed item slopes are class-invariant. This allows for measuring the same latent variables across classes, such that classes only differ concerning the loading structure of items and factors (Alagöz & Meiser, 2024; Kim & Bolt, 2021). Allowing non-fixed item slopes to vary freely would potentially change the meaning of the latent variables across classes. However, item-category intercepts are class-specific in our parameterization. This is because the three classes are likely to differ in their response distributions. As class-specific intercepts can capture different response distributions across classes, modeling intercepts in an unconstrained manner can be assumed to facilitate class separation when estimating the model.

### Differences to Other Faking Mixture Models

Before coming to details about the estimation of the model, we will first delineate how the M-MNRM differs from other faking models including mixture components. Zickar et al. (2004) applied a mixture-distribution IRT model to personality data from an applicant sample. They found three classes characterized by a different ordering and spacing of threshold parameters, which they interpreted as an honest, slight-faking, and extreme-faking class. Nevertheless, since such a mixture modeling approach is fully exploratory, it remains uncertain whether the resulting classes truly capture faking. It may well be that the classes in fact represent other response tendencies. Also, such a model conceptualizes faking only as a discrete variable. Related work, however, emphasized the continuous nature of faking (Ziegler et al., 2015).

Böckenholt (2014; see also Leng et al., 2020) modeled test-takers’ misreporting behavior in sensitive survey questions. This model specifies for every item a binary latent class variable indicating whether a test-taker edits his or her retrieved response. If a test-taker has decided to edit, the selection of desirable response categories is modeled by a transition function. Even though the model is conceptually appealing, it comes with the limitation that only one substantive trait can be modeled. Also, the selection of categories when editing occurs is modeled in a monotonic way, such that the selected categories are assumed to be always higher (lower) if the substantive trait is generally desirable (undesirable).

Brown and Böckenholt (2022) developed a grade-of-membership model to account for intermittent faking. This model assumes that each item response either stems from a “real” (honest) or “ideal” (faking) class that is predicted by a latent editing factor and item characteristics. Within each class, item responses are either a function of substantive traits or a function of a faking factor. Though theoretically elaborate, the model does not include a class where responses are influenced by both substantive traits and faking, which is conceivable considering the findings by Robie et al. (2007) described above. Also, like the approaches by Zickar et al. (2004) and Böckenholt (2014), the model does not explicitly account for nonmonotonic faking effects in a way that, for some items, high faking levels make the selection of non-extreme categories more likely (see Kuncel & Tellegen, 2009; Seitz et al., 2024, 2025).

### Model Estimation

The M-MNRN can be estimated in a Bayesian Markov chain Monte Carlo (MCMC) procedure. Therefore, we implemented the full model (Equation 3), in which the latent regression of class membership on covariates (Equation 8) is nested, in the program *JAGS* (version 4.3.1; Plummer, 2017). We accessed JAGS via the *R* environment (version 4.4.0) using the package *runjags* (Denwood, 2016), and employed the packages *coda* (Plummer et al., 2006) and *MCMCvis* (Youngflesh, 2018) for processing MCMC outputs. The JAGS syntax and *R* code for estimating the model can be found at https://osf.io/vwqf3/.

For model estimation, the following priors were used: Non-fixed item slopes were sampled from a positively-truncated normal distribution ( $\alpha_{\mathrm{id}}\;\sim \;N^{+}(0,\;2^{2})$ ). Class-specific item-category intercepts were drawn from an uncensored normal distribution ( $\gamma_{\mathrm{ikc}}\;\sim \;N(0,\;5^{2})$ ), with the intercept of the first category fixed to 0 due to model identification. Substantive trait and faking scores were drawn from a multivariate normal distribution ( $\theta_{n}\;\sim \;\mathrm{MVN}(\mu ,\;\Sigma )$ ), where μ=0 and Σ was a variance-covariance matrix with unit variances. Thus, covariances represented correlations, which had a uniform prior distribution ( $\rho_{dd^{\prime }}\;\sim \;U(-1,\;1)$ ). Latent regression coefficients were sampled from a normal distribution ( $\beta_{0c},\beta_{\mathrm{pc}}\;\sim \;N(0,\;2^{2})$ ), with coefficients pertaining to the “S&F class” fixed to 0. Class membership was drawn from a categorical distribution ( $\zeta_{n}\;\sim \;\mathrm{Cat}(\pi_{n})$ ), in which $\pi_{n}$ was a vector of person-specific class probabilities resulting from the latent regression of class membership at the respective MCMC iteration.

To obtain point estimates of continuous model parameters, means of posterior distributions were computed. For class membership as a discrete model parameter, the posterior mode was considered (i.e., modal assignment; Dias & Vermunt, 2008). Because of the latent regression of class membership in the M-MNRM, the model does not include explicit class proportion parameters. However, mean class probabilities across persons and MCMC iterations can be calculated to get class proportion estimates. Note that, in the present model, class labels are not arbitrary because different measurement models are explicated for the three classes. This prevents the problem of label switching (Stephens, 2000) that is frequently encountered in the Bayesian estimation of mixture models.

## Simulation Study

To investigate the M-MNRM under different class proportion conditions, we conducted a simulation study analyzing parameter recovery and the model’s superiority over non-mixture models. Also, the simulation should examine whether the correct model (non-mixture vs. mixture) is selected when either one or multiple classes are present in the data.

### Simulation Conditions

The simulation featured 10 class proportion conditions: In Condition 1, class proportions were (33.3%, 33.3%, 33.3%), that is, the three classes were equally sized. Conditions 2 to 4 were conditions in which either the “S&F class” (Condition 2 : (60%, 20%, 20%)), the “S-only class” (Condition 3 : (20%, 60%, 20%)), or the “F-only class” (Condition 4 : (20%, 20%, 60%)) was dominant. In Conditions 5 to 7, one class was absent, either the “S&F class” (Condition 5 : (0%, 50%, 50%)), the “S-only class” (Condition 6 : (50%, 0%, 50%)), or the “F-only class” (Condition 7 : (50%, 50%, 0%)). Conditions 8 to 10 represented data situations with non-mixture populations. That is, only one class was present in the data, either the “S&F class” (Condition 8 : (100%, 0%, 0%)), the “S-only class” (Condition 9 : (0%, 100%, 0%)), or the “F-only class” (Condition 10 : (0%, 0%, 100%)).

### Data Generation and Fitted Models

For every simulation condition, we simulated item responses of a questionnaire measuring 3 substantive traits with 10 items each on a 7-point Likert scale. We chose this simulation design to examine data situations representative of empirical high-stakes datasets (see the empirical demonstration below). To generate the data, we took the following steps:

1. Item slopes $\alpha_{\mathrm{id}}$ : Item slopes of substantive traits and faking were sampled from U(0.5,1) .
2. Scoring weights $s_{\mathrm{idk}}$ : Scoring weight vectors of substantive traits were set to $\left(\begin{array}{ccccccc} 0 & 1 & 2 & 3 & 4 & 5 & 6 \end{array}\right)$ . Scoring weight vectors of faking also had a range from 0 to 6 but varied between items to emulate a situation in which relationships between response categories and desirability are item-specific. Specifically, within every substantive trait scale, scoring weight vectors of faking were generated reflecting monotonically increasing, nonmonotonically increasing, as well as inverted-U-shaped relations between categories and desirability (see Figure 1 in Seitz et al., 2024, for details).
3. Item-category intercepts $\gamma_{\mathrm{ikc}}$ : For every item, the intercept of the first category was fixed to 0. Intercepts of the remaining categories were simulated by sampling sorted thresholds $\tau_{\mathrm{ikc}}$ from U(−2,2) before the resulting values were transformed into cumulative thresholds representing intercepts: $\gamma_{\mathrm{ikc}}=-\sum_{m=0}^{k}\tau_{\mathrm{imc}}$ . This procedure was carried out independently for the three classes.
4. Substantive trait and faking scores $\theta_{\mathrm{nd}}$ : For each of N=1,500 simulated test-takers, three substantive trait scores and a faking score were drawn from MVN(μ,Σ) , with μ=0 and $\Sigma =\left(\begin{array}{cccc} 1 & & & \\ 0 & 1 & & \\ .30 & -.30 & 1 & \\ 0 & .30 & -.30 & 1 \end{array}\right)$ . The assignment of the latent correlations printed in italics to the three substantive trait pairs was randomized between replications.
5. Latent regression coefficients $\beta_{0c},\beta_{\mathrm{pc}}$ : Three covariates of class membership were considered, one with a null effect, one with weak effects, and one with strong effects. With the “S&F class” as the reference class, latent regression coefficients pertaining to this class were fixed to 0. Regression slopes of the null-effect covariate pertaining to the remaining classes were also set to 0, whereas slopes of the weak-effects and strong-effects covariates were sampled from $N(1,\;0.2^{2})$ and $N(2,\;0.2^{2})$ , respectively. Half of the regression slopes within each replication were multiplied by −1 to simulate both positive and negative covariate effects. Regression intercepts were specified to lead to the class proportions of the respective simulation condition.
6. Covariate values $X_{\mathrm{np}}$ : For all simulated test-takers, values on the three covariates were drawn from MVN(μ,Σ) , with μ=0 and Σ as a diagonal matrix with unit variances.
7. Class membership $\zeta_{n}$ : Using Equation (8), class probabilities $\pi_{n}$ were computed for every simulated test-taker, which were then used to sample the actual class membership from $\mathrm{Cat}(\pi_{n})$ .^1^
8. Based on the generated item parameters, substantive trait and faking scores, as well as class memberships, item responses were simulated using Equation (4).
9. Steps 1 to 8 were replicated such that there were 30 generated datasets per condition.

The data generation was performed in *R* using the packages *MASS* (Venables & Ripley, 2002) and *mirt* (Chalmers, 2012). Within each condition, four models were fitted to all generated datasets: a model only accounting for a faking dimension with specified scoring weights (“θ_F_ model”), a model only accounting for substantive traits (“θ_S_ model”), a model accounting for substantive traits and faking (“θ_S_/θ_F_ model”), as well as the M-MNRM accounting for substantive traits and faking with the three latent classes (“mixture θ_S_/θ_F_ model”). Scoring weights were specified as in the data generation. The three non-mixture models were also estimated using the Bayesian estimation framework described above (the non-mixture model syntaxes are also available at https://osf.io/vwqf3/). Four parallel MCMC chains were run for every model. The estimation featured a burnin phase of 2,000 iterations, followed by 5,000 regular iterations.

**Figure 1.**
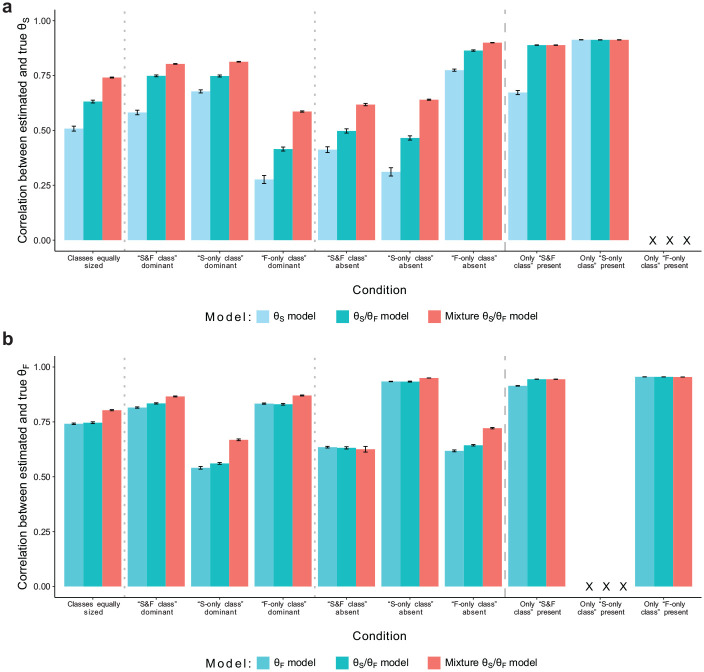
Simulation Study: Recovery of Substantive Trait and Faking Scores. (a) Recovery of Substantive Trait Scores and (b) Recovery of Faking Scores. *Note.* Values reflect the mean correlations (using Fisher’s *z*-transformation) between estimated and true substantive trait scores (a) or faking scores (b) across replications within a condition. Results for substantive traits are aggregated across the three substantive traits used in the simulation. Error bars represent the standard error of the mean. “X” denotes that a proper recovery of the particular parameters is precluded in the respective condition because they have not influenced item responses in the data generation.

### Simulation Results

To check model convergence, we considered $\hat{R}$ values of continuous model parameters (Gelman & Rubin, 1992). These were below 1.1 for all models. Also, we visually inspected MCMC chains of item parameters, latent correlations, and latent regression coefficients and found that trace plots were well-mixed.

#### Model Selection

As mentioned above, we examined if model selection criteria correctly chose the mixture model when there was more than one class in the data, and, crucially, if they chose a non-mixture model when the data came from a non-mixture population. Therefore, we considered the deviance information criterion (DIC; Spiegelhalter et al., 2002) as calculated by Gelman et al. (2004), the widely applicable information criterion (WAIC; Watanabe, 2010), and the leave-one-out information criterion (LOOIC; Vehtari et al., 2017). These measures balance model fit and model parsimony by penalizing the model’s mere fit to the data with the effective number of parameters or by considering out-of-sample predictive accuracy. Table 1 shows the percentages with which DIC, WAIC, and LOOIC selected the correct data-generating model. In conditions with more than one class present in the data, every model selection criterion correctly selected the “mixture θ_S_/θ_F_ model” in all replications. In conditions with only one class in the data, the performance of model selection criteria was still high (above 80%), though in some cases incorrect models were selected. However, incorrect model selections were not only due to an overselection of the mixture model but also due to false model selections within the three non-mixture models. Percentages of incorrect mixture model selections in conditions with only one class were below 10%. It is also important to note that, in replications in which the mixture model was falsely selected, the number of simulated test-takers assigned to a truly non-existent class was negligible (see hit rates below).

**Table 1.** Simulation Study: Percentages of Correctly Selected Models.

Condition	Model selection criterion		
	DIC	WAIC	LOOIC
Classes equally sized	100.0% (100.0%)	100.0% (100.0%)	100.0% (100.0%)
“S&F class” dominant	100.0% (100.0%)	100.0% (100.0%)	100.0% (100.0%)
“S-only class” dominant	100.0% (100.0%)	100.0% (100.0%)	100.0% (100.0%)
“F-only class” dominant	100.0% (100.0%)	100.0% (100.0%)	100.0% (100.0%)
“S&F class” absent	100.0% (100.0%)	100.0% (100.0%)	100.0% (100.0%)
“S-only class” absent	100.0% (100.0%)	100.0% (100.0%)	100.0% (100.0%)
“F-only class” absent	100.0% (100.0%)	100.0% (100.0%)	100.0% (100.0%)
Only “S&F class” present	80.0% (90.0%)	96.7% (100.0%)	90.0% (90.0%)
Only “S-only class” present	83.3% (93.3%)	96.7% (96.7%)	93.3% (96.7%)
Only “F-only class” present	86.7% (93.3%)	93.3% (100.0%)	86.7% (93.3%)

*Note.* Percentages are based on 30 replications per condition. In simulation conditions in which more than one class was present, the “mixture θ_S_/θ_F_ model” was the underlying population model, whereas either the “θ_S_/θ_F_ model,”“θ_S_ model,” or “θ_F_ model” was the population model in conditions in which only the respective class was present. Values in brackets reflect percentages of correct decisions concerning the question of whether a mixture or non-mixture model was the data-generating model. DIC = deviance information criterion; WAIC = widely applicable information criterion; LOOIC = leave-one-out information criterion.

#### Parameter Recovery

To evaluate parameter recovery, we considered the bias of estimation to investigate if parameters were systematically over- or underestimated as well as the root mean square error (RMSE) to investigate the accuracy of estimation. For the recovery of substantive trait and faking scores, we considered the correlation between estimated and true parameters. To examine how well the individual class membership was recovered, we considered the hit rate, which indicates the percentage of persons correctly assigned to their respective class and thus reflects a measure of classification accuracy.

##### Class proportions and class membership

The recovery of class proportions in the “mixture θ_S_/θ_F_ model” is displayed in Table 2. Across conditions, class proportions were estimated with negligible bias and small RMSE. Bias and RMSE did not systematically vary between conditions and the three classes.

**Table 2.** Simulation Study: Recovery of Class Proportions and Hit Rates.

Condition	Class					
	“S&F class”		“S-only class”		“F-only class”	
	Bias (RMSE)	HR	Bias (RMSE)	HR	Bias (RMSE)	HR
Classes equally sized	−0.001 (0.004)	0.975	0.000 (0.002)	0.981	0.000 (0.002)	0.979
“S&F class” dominant	0.000 (0.003)	0.981	0.000 (0.002)	0.981	0.000 (0.002)	0.973
“S-only class” dominant	0.000 (0.002)	0.973	0.000 (0.002)	0.984	0.000 (0.002)	0.978
“F-only class” dominant	−0.001 (0.003)	0.969	0.000 (0.002)	0.982	0.000 (0.003)	0.983
“S&F class” absent	0.002 (0.005)		−0.001 (0.002)	0.985	−0.001 (0.002)	0.984
“S-only class” absent	−0.002 (0.005)	0.980	0.002 (0.004)		0.000 (0.003)	0.982
“F-only class” absent	−0.002 (0.004)	0.981	0.000 (0.002)	0.982	0.002 (0.004)	
Only “S&F class” present	−0.004 (0.009)	0.998	0.002 (0.004)		0.002 (0.005)	
Only “S-only class” present	0.002 (0.004)		−0.003 (0.006)	0.997	0.001 (0.003)	
Only “F-only class” present	0.002 (0.003)		0.001 (0.003)		−0.003 (0.006)	0.998

*Note.* Values reflect the mean bias and RMSE (in brackets) of estimated class proportions across replications within a condition, as well as HRs. HRs are the mean percentages of simulated test-takers correctly assigned to their respective class. HR = hit rate; RMSE = root mean square error.

Apart from an accurate estimation of overall class proportions, individual class membership was also recovered well, indicated by high hit rates (see Table 2). In conditions with more than one class in the data, hit rates ranged from 96.9% to 98.5%. In conditions with only one class, hit rates were close to 100%. That is, virtually no simulated test-taker was assigned to a class that was empty in the data generation, which gives another indication along with model selection criteria that a less complex (i.e., a non-mixture) model should be used in this case (see also the discussion below).

##### Latent regression coefficients

Similar to the recovery of class proportions, the “mixture θ_S_/θ_F_ model” estimated latent regression coefficients without systematic bias (see Table 3). This applied to regression intercepts as well as regression slopes representing the covariate effects. RMSE was also small for both intercepts and slopes. Concerning intercepts, RMSE was relatively more pronounced in conditions with unequally sized classes compared to conditions with equal class proportions and conditions with one class being absent. Concerning slopes, RMSE increased slightly with stronger covariate effect sizes.

**Table 3.** Simulation Study: Recovery of Latent Regression Coefficients.

Condition	Bias (RMSE) of regression coefficients			
	Intercepts	Slopes of null-effect covariate	Slopes of weak-effects covariate	Slopes of strong-effects covariate
Classes equally sized	0.00 (0.09)	0.00 (0.07)	−0.03 (0.09)	0.02 (0.13)
“S&F class” dominant	−0.01 (0.11)	0.01 (0.08)	0.00 (0.10)	0.01 (0.11)
“S-only class” dominant	−0.02 (0.15)	−0.01 (0.07)	−0.01 (0.11)	0.03 (0.14)
“F-only class” dominant	0.00 (0.13)	0.01 (0.08)	0.00 (0.09)	−0.02 (0.13)
“S&F class” absent	−0.01 (0.06)	0.02 (0.07)	−0.03 (0.11)	0.03 (0.18)
“S-only class” absent	0.03 (0.06)	0.01 (0.06)	0.01 (0.07)	0.00 (0.09)
“F-only class” absent	0.00 (0.05)	0.01 (0.06)	0.02 (0.07)	0.01 (0.08)

*Note.* Values reflect the mean bias and RMSE (in brackets) of estimated latent regression coefficients across replications within a condition. In the condition in which the “S&F class” was absent, the “S-only class” is treated as the reference class. Results for conditions with only one class in the data are left out because class membership is a constant in these conditions, which precludes a proper recovery of regression coefficients. RMSE = root mean square error.

##### Substantive trait and faking scores

Figure 1a shows the recovery of substantive trait scores in models including substantive trait dimensions. The overall level of parameter recovery primarily varied with the proportion of simulated test-takers for whom item responding was not influenced by substantive traits. Within conditions, the “θ_S_/θ_F_ model” generally exhibited higher correlations between estimated and true substantive trait scores than the “θ_S_ model.” Crucially, the “mixture θ_S_/θ_F_ model” improved correlations further in conditions in which more than one class was present in the data. In conditions with only one class, the “mixture θ_S_/θ_F_ model” and “θ_S_/θ_F_ model” did not differ.

A similar pattern emerged for the recovery of faking scores (see Figure 1b). The “mixture θ_S_/θ_F_ model” yielded the highest correlations between estimated and true faking scores in most conditions with more than one class, whereas the “θ_S_/θ_F_ model” and “θ_F_ model” differed only slightly. In the condition in which the “S&F class” was absent, all three models exhibited comparable levels of correlation. Again, in conditions with only one class, the “mixture θ_S_/θ_F_ model” and “θ_S_/θ_F_ model” performed equivalently.

##### Item slopes

**Figure 2.**
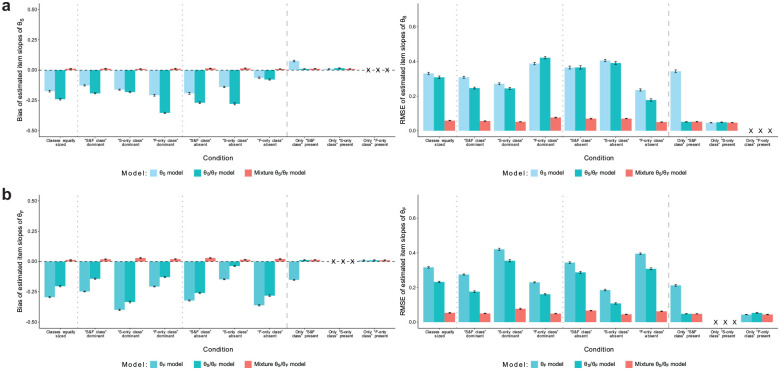
Simulation Study: Recovery of Item Slopes. (a) Recovery of Item Slopes of Substantive Traits and (b) Recovery of Item Slopes of Faking. *Note.* Values reflect the mean bias or RMSE of estimated item slopes of substantive traits (a) or faking (b) across replications within a condition. Error bars represent the standard error of the mean. “X” denotes that a proper recovery of the particular parameters is precluded in the respective condition because they have not influenced item responses in the data generation. RMSE = root mean square error.

The recovery of item slopes is presented in Figure 2. Substantive trait slopes (Figure 2a) were generally underestimated in the “θ_S_ model” and “θ_S_/θ_F_ model” and had pronounced RMSE, unless the respective model matched the population model in conditions with only one class in the data. The “mixture θ_S_/θ_F_ model” eliminated the bias in all conditions and reduced RMSE considerably. Likewise, estimates of faking slopes (Figure 2b) in the “θ_F_ model” and “θ_S_/θ_F_ model” were negatively biased when there was more than one class in the data, whereas the “mixture θ_S_/θ_F_ model” showed negligible bias. RMSE was also much smaller in the mixture model than in the non-mixture models. In conditions with only one class, the mixture model and the correctly specified non-mixture model both yielded unbiased estimates with equivalent RMSE.

##### Item-category intercepts

**Figure 3.**
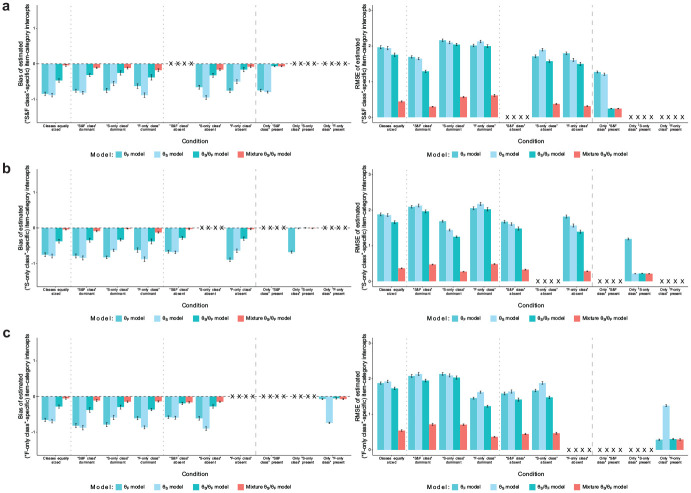
Simulation Study: Recovery of Item-Category Intercepts. (a) Recovery of Item-Category Intercepts of the “S&F Class”; (b) Recovery of Item-Category Intercepts of the “S-only Class”; and (c) Recovery of Item-Category Intercepts of the “F-only Class.” *Note.* Values reflect the mean bias or RMSE of estimated item-category intercepts with respect to the true values in the “S&F class” (a), “S-only class” (b), or “F-only class” (c) across replications within a condition. For the mixture model, the respective class-specific intercept estimates are considered. Error bars represent the standard error of the mean. “X” denotes that a proper recovery of the particular parameters is precluded in the respective condition because they have not influenced item responses in the data generation. RMSE = root mean square error.

Figure 3 shows the recovery of item-category intercepts, which were class-specific in the data generation. The three non-mixture models generally yielded negatively biased intercept estimates with pronounced RMSE when the data consisted of more than one class. Only in conditions with just one class and if the respective model was not underparameterized, the non-mixture models estimated intercepts without bias and with small RMSE. In contrast, the mixture model’s class-specific intercept estimates had very small to negligible bias and RMSE in all conditions.

##### Latent correlations

**Figure 4.**
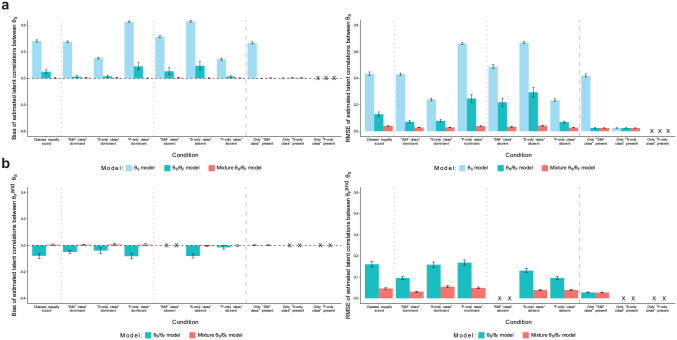
Simulation Study: Recovery of Latent Correlations. (a) Recovery of Latent Correlations Between Substantive Traits and (b) Recovery of Latent Correlations Between Faking and Substantive Traits. *Note.* Values reflect the mean bias or RMSE of estimated latent correlations between substantive traits (a) or faking and substantive traits (b) across replications within a condition. Results are aggregated across the three substantive traits used in the simulation. Error bars represent the standard error of the mean. “X” denotes that a proper recovery of the particular parameters is precluded in the respective condition because they have not influenced item responses in the data generation. RMSE = root mean square error.

Latent correlations between substantive traits (see Figure 4a) were heavily overestimated in the “θ_S_ model,” especially in conditions with smaller proportions of simulated test-takers for whom item responding was influenced by substantive traits. The “θ_S_/θ_F_ model” considerably reduced this bias, even yielding fully unbiased estimates in conditions with a small “F-only class” proportion. The “mixture θ_S_/θ_F_ model,” however, estimated latent correlations between substantive traits without bias in all conditions. Also, RMSE was smaller in the mixture model than in the two non-mixture models. Regarding latent correlations between faking and substantive traits (see Figure 4b), the “θ_S_/θ_F_ model” generally yielded underestimates with considerable RMSE, whereas the “mixture θ_S_/θ_F_ model” afforded an unbiased estimation with smaller RMSE. In the condition in which only the “S&F class” was present, both models exhibited no bias and equivalent RMSE.

## Empirical Demonstration

Along with the reported simulation study, we applied the M-MNRM to three empirical datasets from personnel selection contexts. This allowed us to examine the existence and prevalence of the three latent faking classes in high-stakes assessment data from different job application contexts.

### Datasets

The datasets were made available by a Germany-based testing company that develops psychological tests for personnel selection. All three datasets contained responses from test-takers who had taken a personality test as part of an application for a job. Dataset 1 consisted of N=3,046 test-takers applying for a bank apprenticeship at a financial institution in Germany (gender: 60.4% female, 39.6% male; age: M=18.22 years, SD=1.98 , range=[14,29] ; this dataset was also analyzed by Seitz et al., 2025). Dataset 2 comprised N=1,824 applicants for a police officer traineeship at a German police department (gender: 30.0% female, 70.0% male; age: M=21.02 years, SD=4.61 , range=[15,39] ). Dataset 3 included N=2,007 test-takers who had applied for a position as insurance agent at a Germany-based insurance company (gender: 28.7% female, 71.2% male).^2^

In the present empirical demonstration, we modeled data from three substantive trait scales that were available in all datasets. These scales assessed the personality traits of Emotional Stability, Extraversion, and Conscientiousness. Emotional Stability was measured with 12 items (Dataset 1: Cronbach’s α=.75 ; Dataset 2: α=.74 ; Dataset 3: α=.70 ), Extraversion with 9 items (Dataset 1: α=.74 ; Dataset 2: α=.67 ; Dataset 3: α=.72 ), and Conscientiousness with 10 items (Dataset 1: α=.79 ; Dataset 2: α=.77 ; Dataset 3: α=.66 ). Item responses were given on a 7-point Likert scale (0 = *does not apply at all* to 6 = *applies fully*). Along with these three substantive trait scales, covariates were available in the datasets, namely, the score of an integrity test, the score of an achievement motivation test, and a measure of intelligence (aggregate of verbal, numeric, and figural cognitive ability test scores).

### Pilot Studies

Before fitting models to the data, we ran a series of pilot studies to determine scoring weights for the faking dimension in the different personnel selection settings. Therefore, we asked independent samples of participants to rate the social desirability of every response category of every item with respect to the three job application settings (Seitz et al., 2025; see also Kuncel & Tellegen, 2009). Participants should hereby put themselves into the perspective of a person who is currently applying for the respective job, and rate desirability accordingly. The Supplemental Material provides details on the procedure, samples, and results of the pilot studies.

### Results of the Empirical Demonstration

We fitted the same four models as in the simulation to all three datasets. Regarding scoring weights, we specified values as in Equation (2) and used the job-specific mean desirability ratings from the pilot studies as scoring weights of faking, which we linearly transformed to a possible range from 0 to 6 to achieve a common metric of scoring weights across dimensions. Again, we used JAGS through the *R* environment to estimate the models. However, for the empirical analyses, we estimated every model by running 12 parallel MCMC chains that featured 15,000 iterations each, with the first 5,000 iterations discarded as burnin.^3^ $\hat{R}$ values of continuous model parameters were all below 1.1, indicating that all models converged. Also, by visual inspection, we found well-mixed trace plots of item parameters, latent correlations, and latent regression coefficients.

#### Model Selection and Model Fit

Table 4 contains fit indices of the four estimated models. The pattern of results was consistent across the three datasets. Regarding relative fit, DIC, WAIC, and LOOIC all selected the “mixture θ_S_/θ_F_ model,” indicating that the mixture model in all datasets yielded a better compromise between fit and parsimony than the three non-mixture models. Regarding absolute fit, we used posterior predictive model checking (PPMC; e.g., Sinharay et al., 2006), which involves simulating data based on the model parameters’ posterior distribution and comparing the simulated data to the observed data. As a measure of misfit, we considered the standardized root mean square residual (SRMR), which indicates the discrepancy between the model-implied and observed item intercorrelations.^4^ The “θ_F_ model” had the largest SRMR in all job application contexts, followed by the “θ_S_ model” and “θ_S_/θ_F_ model” (see Table 4). Crucially, the “mixture θ_S_/θ_F_ model” consistently yielded the smallest SRMR, indicating that the mixture model had the best absolute fit in the three datasets.

**Table 4.** Empirical Demonstration: Model Fit Indices.

Model	Model selection criterion			SRMR
	DIC	WAIC	LOOIC	
Dataset 1 (bank apprenticeship)				
“θ_F_ model”	273,890.3	272,099.5	273,791.2	0.136
“θ_S_ model”	260,497.4	254,836.5	258,927.7	0.076
“θ_S_/θ_F_ model”	256,858.1	249,733.1	254,442.7	0.066
**“Mixture θ_S_/θ_F_ model”**	**254,427.1**	**246,881.8**	**251,235.1**	**0.056**
Dataset 2 (police officer traineeship)				
“θ_F_ model”	158,887.7	157,312.9	158,261.5	0.142
“θ_S_ model”	150,546.9	146,721.1	149,101.2	0.101
“θ_S_/θ_F_ model”	146,888.4	142,359.9	144,863.4	0.064
**“Mixture θ_S_/θ_F_ model”**	**145,447.3**	**140,678.5**	**142,999.4**	**0.059**
Dataset 3 (position as insurance agent)				
“θ_F_ model”	164,408.8	163,075.1	163,994.7	0.126
“θ_S_ model”	157,679.1	153,093.9	155,437.5	0.121
“θ_S_/θ_F_ model”	150,547.5	145,811.4	148,618.3	0.072
**“Mixture θ_S_/θ_F_ model”**	**146,579.0**	**141,219.6**	**143,669.9**	**0.061**

*Note.* Dataset 1: N=3,046 ; Dataset 2: N=1,824 ; Dataset 3: N=2,007 . The best-fitting model within each dataset is printed in bold. DIC = deviance information criterion; WAIC = widely applicable information criterion; LOOIC = leave-one-out information criterion; PPMC = posterior predictive model checking; SRMR = standardized root mean square residual (based on PPMC).

#### Class Proportions

Looking at the estimated class proportions in the “mixture θ_S_/θ_F_ model,” we found that every class had a considerable size in all datasets (see Table 5). Classes were, however, not equally sized. In all datasets, the “S&F class” had the largest class proportion (46.8–57.4%), the “S-only class” was the second largest class (27.0–44.0%), and the “F-only class” made up the smallest class (9.2–15.6%). Estimates of class proportions did not differ much between the three job application contexts.

**Table 5.** Empirical Demonstration: Estimated Class Proportions and Model Entropies.

Dataset	Class			Entropy
	“S&F class”	“S-only class”	“F-only class”	
Dataset 1 (bank apprenticeship)	0.520 [0.468, 0.572]	0.384 [0.345, 0.423]	0.096 [0.077, 0.117]	0.854
Dataset 2 (police officer traineeship)	0.468 [0.407, 0.529]	0.440 [0.398, 0.482]	0.092 [0.068, 0.118]	0.892
Dataset 3 (position as insurance agent)	0.574 [0.514, 0.632]	0.270 [0.232, 0.310]	0.156 [0.125, 0.190]	0.900

*Note.* Dataset 1: N=3,046 ; Dataset 2: N=1,824 ; Dataset 3: N=2,007 . Values in brackets represent the 95% credible interval.

Additionally, we examined classification diagnostics of the “mixture θ_S_/θ_F_ model,” namely, posterior class probabilities and model entropy. These diagnostics give an indication about the (un)certainty of class assignments in mixture models (Masyn, 2013). In terms of posterior class probabilities, we considered for each test-taker the percentage with which the modal class (i.e., the estimated class of a test-taker) was sampled during the MCMC estimation. The mean posterior class probabilities across test-takers were high for the three classes in all datasets (Dataset 1: 93.1–94.2%; Dataset 2: 94.7–95.5%; Dataset 3: 94.0–96.8%). Posterior class probabilities ( $\pi_{\mathrm{nc}}$ ) can be condensed in the measure of entropy, which has a range from 0 to 1 and is computed as $1-\frac{\sum_{n=1}^{N}\sum_{c=1}^{3}(-\pi_{\mathrm{nc}\;}\log(\pi_{\mathrm{nc}}))}{N\;\log(3)}$ for the described mixture model. As noted in Table 5, entropies were also high in all datasets (0.854–0.900; cf. Vermunt, 2010).

#### Latent Regression of Class Membership

As mentioned above, the datasets also contained covariates. We included these variables as latent predictors of class membership in the “mixture θ_S_/θ_F_ model” in all datasets. Table 6 shows the estimates of latent regression slopes. Results differed slightly between the datasets but were in general consistent. Higher integrity scores were associated with higher probabilities of being a member of the “S-only class” compared to being a member of the “S&F class” (i.e., the reference class). At the same time, higher integrity scores were generally associated with lower probabilities of being an “F-only class” member compared to being an “S&F class” member. For achievement motivation, results were inconsistent. Both among applicants for a bank apprenticeship and among applicants for a position as insurance agent, achievement motivation scores did not consistently predict class membership. Among applicants for a police officer traineeship, however, achievement motivation was negatively related to being an “S-only class” member and positively related to being an “F-only class” member. Similar to integrity, test-takers with higher scores of intelligence were more likely to belong to the “S-only class” compared to the “S&F class,” whereas higher intelligence was associated with a lower probability of belonging to the “F-only class” compared to the “S&F class.”

**Table 6.** Empirical Demonstration: Estimated Latent Regression Slopes.

	Dataset		
	Dataset 1 (bank apprenticeship)	Dataset 2 (police officer traineeship)	Dataset 3 (position as insurance agent)
“S-only class” vs. “S&F class” (reference class)			
Integrity	1.45 [1.21, 1.71]	0.75 [0.47, 1.05]	0.38 [0.13, 0.65]
Achievement motivation	0.29 [0.10, 0.49]	−1.16 [−1.43, −0.91]	0.08 [−0.29, 0.46]
Intelligence	1.31 [1.13, 1.48]	0.75 [0.56, 0.94]	0.59 [0.43, 0.75]
“F-only class” vs. “S&F class” (reference class)			
Integrity	−1.13 [−1.56, −0.71]	−0.65 [−1.07, −0.22]	−0.18 [−0.47, 0.12]
Achievement motivation	0.00 [−0.32, 0.34]	0.89 [0.59, 1.19]	0.00 [−0.42, 0.42]
Intelligence	−1.39 [−1.65, −1.14]	−0.59 [−0.85, −0.34]	−0.36 [−0.52. −0.20]

*Note.* Dataset 1: N=3,046 ; Dataset 2: N=1,824 ; Dataset 3: N=2,007 . All predictors were *z*-standardized. Positive slopes indicate that higher predictor values go along with higher probabilities of being a member of the “S-only class” or “F-only class” compared to the “S&F class.” Values in brackets represent the 95% credible interval.

### Validation of Class Assignments

#### Response Distributions Within Classes

**Figure 5.**
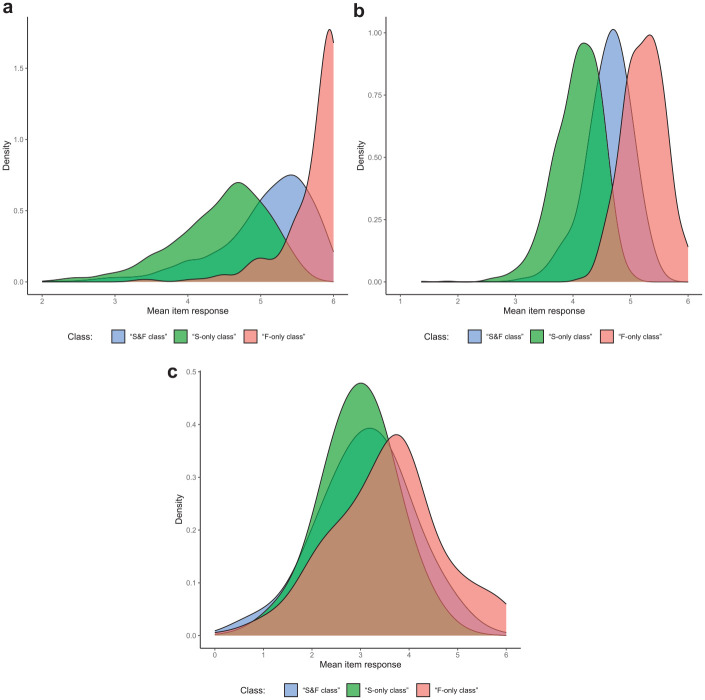
Empirical Demonstration: Class-Specific Distributions of Mean Item Responses for Items with Different Desirability Characteristics. (a) Items With a Highest-Desirability Category of “6”; (b) Items With a Highest-Desirability Category of “5”; (c) Items With a Highest-Desirability Category of “2,”“3,” or “4.” *Note.* Exemplary illustration for Dataset 1 (job application for a bank apprenticeship). The pattern for the other datasets was analogous. Plots display kernel densities of mean item responses, split by the three classes test-takers were assigned to by the “mixture θ_S_/θ_F_ model” (“S&F class”: n=1,597 , “S-only class”: n=1,161 , “F-only class”: n=288 ). (a) Depicts the distributions for items at which the response category with highest desirability in the pilot study was “6,” (b) for items with a highest-desirability category of “5,” and (c) for items with a highest-desirability category of “2,”“3,” or “4” (see the Supplemental Material for more information on the pilot study).

To investigate whether the classes indeed represented the response strategies outlined above, we analyzed response distributions within the three classes. For items at which the highest response category is most desirable, one can expect test-takers responding solely based on a faking dimension (i.e., the “F-only class”) to yield higher mean responses than test-takers considering substantive traits and faking (i.e., the “S&F class”). Test-takers only responding based on substantive traits (i.e., the “S-only class”) should in turn yield the lowest mean responses. However, for items at which desirability does not increase monotonically with higher response categories, different effects can be expected. For items having their category of highest desirability above the midpoint though not at the extreme of the rating scale, differences in mean responses between the classes should be less pronounced compared to items at which the highest category is most desirable. In contrast, there should be no substantial mean differences between classes for items having their highest-desirability category at the scale midpoint. Figure 5 shows the class-specific distributions of mean item responses for the exemplary case of Dataset 1 (the pattern for the other datasets looked very similar). Results were generally in line with expectations, which supports the plausibility of class assignments in the “mixture θ_S_/θ_F_ model.”

#### Class Assignment of New Cases

To provide further evidence that the M-MNRM can afford a valid classification of a test-taker’s response strategy based on his or her response pattern, we applied the fitted “mixture θ_S_/θ_F_ model” to another dataset that was made available by the testing company. This dataset consisted of N=306 subjects (gender: 49.7% female, 48.4% male; age: M=35.05 years, SD=11.61 , range=[18,64] ) who responded to the same personality items used in our empirical analyses above. However, responses were not given in a hiring setting but in the context of career counseling. The personality test was embedded in a series of assessments based on which subjects received suggestions regarding suitable jobs and vocational paths. That is, one can expect that a considerably smaller proportion engages in faking in such a context, because honest responding is essential for effective career counseling and because responses are not used for selection purposes.

For this validation analysis, we fixed item parameters and latent correlations to the estimated values from the “mixture θ_S_/θ_F_ model” in Dataset 3 (see Wetzel et al., 2021, for a similar approach),^5^ and estimated the class proportions in the career counseling dataset. Because the covariates from the high-stakes datasets were not part of the career counseling assessment, we did not include any predictors in the latent regression of class membership. The estimated class proportions were 18.5% for the “S&F class” (95% credible interval: [14.1%, 23.4%]), 80.7% for the “S-only class” [75.8%, 85.1%], and 0.8% for the “F-only class” [0.1%, 2.1%]. That is, compared to the high-stakes context, the model assigned a much smaller proportion of subjects to classes including a faking dimension. The majority of subjects were instead classified as following a response strategy without faking, which is in line with expectations.

## General Discussion

In this article, we proposed a mixture modeling extension of the MNRM to account for qualitatively different response strategies in high-stakes personality assessments. In the non-mixture MNRM, all test-takers are assumed to engage in some degree of faking, which influences item responses along with substantive traits (Seitz et al., 2024, 2025). Faking is represented as a quantitative difference variable in this model. In our mixture extension, however, faking is modeled in terms of both a continuous and discrete variable. This is in line with Kiefer and Benit’s (2016) conclusion that a combined use of quantitative and qualitative modeling techniques would fit the current understanding of faking behavior best (cf. also Ziegler et al., 2015). In the M-MNRM, the discrete nature of faking is modeled by a latent class that represents conceivable response strategies in high-stakes assessments. The continuous nature of faking is modeled by a quantitative latent variable that represents the degree of aligning responses with desirability. Importantly, as opposed to the non-mixture MNRM, the M-MNRM is parameterized such that the quantitative faking variable only influences item responses if a test-taker adheres to a measurement model that includes a faking dimension.

### Summary of Results

In the simulation study, we evaluated the M-MNRM in terms of parameter recovery compared to alternative non-mixture models. Overall, we found the M-MNRM to be superior to models without mixture components when there are indeed multiple classes in the data. Four points are worth emphasizing: First, parameter recovery in the M-MNRM was fairly stable across the different class proportion conditions. That is, the M-MNRM does not seem to require roughly equal class sizes but can outperform non-mixture models also if one class is much larger than the other classes or if one class is completely absent. Second, classification accuracy of the M-MNRM was high in all conditions, indicating that the model can correctly categorize response patterns as stemming from one of the described response strategies. Third, along with a better recovery of item parameters and latent correlations, the M-MNRM also improved the estimation of individual substantive trait scores, as the estimation of individual person parameters is based on a class-specific measurement model.^6^ Fourth, covariate effects of different sizes were recovered well by the M-MNRM, such that substantive relationships between response strategy use and variables of interest can be modeled and tested effectively.^7^

At the same time, when there is only one class in the data, we found that the M-MNRM is not inferior to the non-mixture model representing the underlying population model. That is, although overparameterized, the M-MNRM does not seem to introduce bias or afford less precise estimates in non-mixture populations. This can be explained by the fact that the M-MNRM fitted to a non-mixture population assigns virtually all test-takers to the respective single class, such that the same set of data is used for parameter estimation in both the mixture and non-mixture model. Nevertheless, the M-MNRM constitutes an overly complex model when there is only one class in the data. Hence, for the sake of model parsimony and to reduce the risk of overfitting, a non-mixture model should be preferred in this case. Researchers and practitioners have two options to decide which model to choose in a given dataset: First, formal model selection criteria (DIC, WAIC, LOOIC) can be used. As opposed to model comparisons using likelihood-ratio tests in frequentist settings, DIC, WAIC, and LOOIC are no significance tests indicating whether a model fits the data significantly better than a more parsimonious model. Instead, they are information criteria that quantify the balance between mere model fit and model parsimony, which is achieved by penalizing model fit with the effective number of parameters or by considering out-of-sample predictive accuracy. As information criteria are descriptive measures, there are no fixed cutoffs or rules of thumb for differences in DIC, WAIC, or LOOIC to be considered meaningful. Instead, common practice is to simply select the model with the lowest information criterion value. In the simulation, when the population model was a mixture model, information criteria correctly chose the mixture model in all replications; when the population model was a non-mixture model, information criteria correctly chose a non-mixture model in more than 90% of the replications. Considering information criteria can hence be a trustworthy approach for choosing between models. Second, researchers and practitioners can consider the estimated class sizes in the M-MNRM for model selection. As indicated by the simulation, the M-MNRM can accurately estimate class proportions and test-takers’ class membership under different kinds of data-generating models. Importantly, the simulation showed that this is also the case when one or two classes are truly absent in the data, as the proportion of empty classes is then indeed estimated to be 0 (see Alagöz & Meiser, 2024; Kim & Bolt, 2021; Tijmstra et al., 2018, who observed the same result for other mixture models estimated in a Bayesian framework). Given that DIC, WAIC, and LOOIC occasionally yielded false model selections in the simulation, we advise researchers and practitioners to pay close attention to how many test-takers actually make up each class, and let this information guide the decision of which model to choose: If test-takers are distributed across all classes, the full M-MNRM is appropriate; if one class is virtually empty, it is appropriate to not include the particular class; and if a single class contains virtually all test-takers, the respective non-mixture model is appropriate.

In the empirical demonstration, we showed that the M-MNRM can also prove successful in real high-stakes assessment data. We found the M-MNRM to be selected over non-mixture models in three datasets from different job application contexts, despite its higher complexity in terms of additional parameters. Also with regard to absolute fit, PPMC analyses revealed that the M-MNRM could describe the data better than non-mixture models. Furthermore, entropy values indicated good class separation in all empirical datasets. Comparing the results of the M-MNRM between the three job application contexts, it should be noted that there were no pronounced differences. Estimates of class proportions, for instance, were fairly constant in all datasets, suggesting that the non-mixture MNRM is misspecified for about 50% of test-takers in high-stakes assessments. For approximately 40%, a measurement model including only substantive traits seems to be more appropriate, whereas a one-dimensional measurement model of faking seems to describe the response behavior best for about 10%. Moreover, class membership was consistently predicted by integrity and intelligence, such that test-takers in the “S-only class” had the highest integrity and intelligence values and test-takers in the “F-only class” had the lowest values. Validation analyses also provided evidence for the plausibility of empirical class assignments performed by the M-MNRM, as class-specific response distributions and class proportions in a low-stakes sample were in line with expectations. To sum up, the consistent results across the three independent samples of job applicants provide evidence for the general usefulness of the M-MNRM in high-stakes personality assessments.

### Utility of the Model

As mentioned in the introduction of the model, the M-MNRM constitutes a confirmatory as opposed to an exploratory mixture model. Jeon (2019) argued that, whereas exploratory mixture models allow researchers to *explore* the potential presence and substantive nature of multiple latent classes in the data, confirmatory mixture models are suited to *confirm* the existence and attributes of hypothesized latent classes. Similar to the distinction between exploratory and confirmatory factor models, the confirmatory approach in mixture modeling comes with stronger assumptions in the form of a-priori-constrained parameters. However, this precise definition of classes (a) allows for a theory-driven modeling of the data, (b) facilitates the interpretability of results, and (c) alleviates the risk of exploiting noise in the data (i.e., overfitting; cf. Celeux et al., 2019; van Havre et al., 2015). In our case, we modeled the three conceptual response strategies of how test-takers may or may not consider substantive traits and faking (Robie et al., 2007) and found all three classes to be present in empirical high-stakes data despite their restrictive definition with item slopes of non-used dimensions fixed to 0.

In applied settings, the combination of qualitative and quantitative modeling techniques allows practitioners (a) to make individual classifications regarding the response strategy of test-takers and (b) to more properly estimate and report substantive trait scores. In terms of individual response strategy classifications, the M-MNRM offers a faking detection technique that is more sophisticated than other indirect methods for detecting faking (e.g., LaHuis & Copeland, 2009; Sun et al., 2022; see Goldammer et al., 2024) or lie scales (e.g., Paulhus, 1988). Accurate faking classifications are vital for personality assessments used in high-stakes contexts. For instance, if test-takers have a very high probability of belonging to the “F-only class,” decision-makers should be informed that classical test scores, such as sum scores of raw item responses, are likely no valid indicators of the intended-to-be-measured traits. Likewise, accurate classifications are essential for the estimation of substantive trait scores in the two remaining classes to be based on the correct measurement model. As the simulation showed, the M-MNRM can indeed afford high hit rates and, consequently, improve the estimation of substantive trait scores compared to non-mixture models (more information on the class-specific estimation of person parameters can be found in the Supplemental Material).

### Limitations and Future Research Directions

Some limitations as well as future research directions warrant mentioning. One caveat concerns the direct comparability of person parameter estimates across classes, which can be an issue in all types of latent class models. To be able to make meaningful comparisons of person parameters across classes, parameters must be on a common scale (Paek & Cho, 2015). In the M-MNRM as presented in this article, item slopes of dimensions not fixed to 0 are constrained to be class-invariant, in order to allow for the same latent variables to be measured in every class. Item-category intercepts, however, are unconstrained between classes, in order to capture different response distributions in the three classes, which should facilitate class separation when estimating the model. There are different approaches for establishing a common scale of person parameters across classes despite this non-invariance of intercepts. These resemble scaling methods in the context of test equating (see Kolen & Brennan, 2004) as well as approaches for creating a common scale of item difficulty parameters in mixture Rasch models (Paek & Cho, 2015; Rost, 1990). One option is to model a set of so-called anchor items. Anchor items are items whose slope and intercept parameters are the same in all classes, such that these items create a common scale of person parameters. In the context of faking, items with neutral social desirability could serve as viable anchor items. Alternatively, assuming that the response strategies test-takers employ in a high-stakes assessment do not transfer to an assessment context where stakes are low, items administered to test-takers in a low-stakes assessment setting could be candidate anchor items. If no anchor items are available (as in our empirical demonstration), a common scale of person parameters can also be achieved if the latent classes follow the same true trait distribution. For model identification, latent means and variances of the multivariate normal distribution of person parameters are set to 0 and 1, respectively, for all classes in the M-MNRM. If this class-invariant identification of the scale of person parameters is in line with the truth (i.e., if classes truly do not differ in their trait distribution), person parameter estimates should be on the same scale across classes and hence be comparable. Such a situation was modeled in an additional simulation reported in the Supplemental Material, where intercepts were systematically different between classes but person parameters were drawn from the same multivariate normal distribution irrespective of class membership. As illustrated in Figure S2, non-mixture models in such a scenario yield pronounced mean differences between classes in substantive trait score estimates. In contrast, the M-MNRM does not produce such a bias, allowing that test-takers can be meaningfully ranked across classes on substantive traits.^8^ The assumption of a homogeneous trait distribution across classes can be appropriate for many personality constructs. However, if this assumption is violated, a common scale of person parameters will not be achieved unless the scale is matched by other test equating methods (such as anchor items; see Kolen & Brennan, 2004). Hence, we generally advise researchers and practitioners to compare person parameters of test-takers from different classes with caution.

Another set of limitations is related to the simulation of the current article, which featured different class proportion conditions but was limited to a fixed sample size and test length. Even though our simulation design was representative of datasets in empirical high-stakes settings (see the empirical demonstration above), future research should study the performance of the M-MNRM in data situations with different numbers of test-takers, items, and substantive trait scales. Also, even though monotonically increasing, nonmonotonically increasing, as well as inverted-U-shaped relations between response categories and desirability were simulated, scoring weights of faking were equidistant within the segments of the desirability trajectories in our simulation (see Figure 1 in Seitz et al., 2024). In empirical settings, however, the relation between categories and desirability may well take on idiosyncratic forms (Kuncel & Tellegen, 2009). To assess the sensitivity of the M-MNRM to situations where categories are related to desirability in idiosyncratic, non-equidistant ways, we ran an additional simulation in which we set scoring weights of faking to desirability values collected in the pilot study for Dataset 1 of the empirical demonstration (see the Supplemental Material for details). This should emulate realistic relations between categories and desirability, where scoring weights of faking are not necessarily equidistant. Results of this additional simulation were essentially the same as in the main simulation with equidistant scoring weights of faking. Nevertheless, future research could examine the M-MNRM’s sensitivity to different kinds of item desirability characteristics in more detail (cf. Seitz et al., 2024, who conducted a similar investigation in the context of the non-mixture MNRM). Additionally, the number of replications per condition in our simulation was limited to 30. This was primarily due to the computational complexity of the M-MNRM (the estimation of the model in a single replication of the simulation took more than ten hours on a high-performance computer). As indicated by the error bars in Figures 1 to 4, results were nevertheless fairly reliable with 30 replications per condition. The model’s computational complexity with its Bayesian estimation may in itself constitute a limiting factor for applied researchers and practitioners who wish to apply the model. However, this limitation will be alleviated in the future with the ever-increasing availability of high-performance computing machines. To facilitate dissemination of the model, we provide commented syntax files for estimating the M-MNRM in an exemplary dataset (available at https://osf.io/vwqf3/).

Also, notwithstanding the above-described positive features of confirmatory mixture models, we encourage future research to model different response strategies in high-stakes assessments in a less restrictive manner, for instance, by specifying class- and dimension-specific proportionality constants on a class-invariant matrix of item slopes. Future studies could also test alternative parameterizations of the “F-only class,” for example, one with a model of independence. In such a model, “F-only” test-takers’ overall tendency to respond according to desirability characteristics would only be captured by item-category intercepts, whereas no variance would be explained by a common factor in this class (i.e., all variation would be unsystematic). However, it might be difficult to separate such a class from a class of test-takers who simply respond inattentively (cf. Jin et al., 2018), even though careless responding is arguably very rare in high-stakes assessments. An additional extension of the model would be to allow class membership to vary not only between persons but also between items. This would further increase the flexibility of the model and account for switches between response strategies over the course of the questionnaire. However, it should be noted that the information for class assignments in such a person-by-item mixture model would be very sparse compared to a person mixture model (namely, single item responses instead of a whole response vector). To overcome this challenge, it could be worthwhile incorporating external information, such as response times or other process data (see Ulitzsch et al., 2022, who modeled careless responding with a person-by-item mixture model that included response times). In such a model, item-level covariates like item wording or other item characteristics could be modeled to examine which types of item content are particularly susceptible to faking.

Furthermore, it would be appealing to compare the faking detection accuracy of the M-MNRM to the accuracy of other recent faking detection methods, such as machine-learning-based approaches (Calanna et al., 2020; see also Nie et al., 2025) or approaches using IRTree models (Sun et al., 2022). Seeing under which conditions the different methods perform best could help develop an integrative faking detection technique that combines features from mixture IRT(ree) modeling and machine learning. Another interesting endeavor for future studies analyzing follow-up data from hired applicants would be to link test-takers’ class membership in the M-MNRM to real-world job performance outcomes, especially to contextual performance and counterproductive work behavior. In the empirical demonstration of the current article, class membership was associated with integrity, achievement motivation, and intelligence. If, however, membership in the “S-only class” was related to actual organizational citizenship behavior, or if membership in the “F-only class” predicted undesired actions like turnover or absenteeism, distinguishing between different response strategies in high-stakes assessments would not only be important from a psychometric measurement perspective but also provide in itself a valuable piece of information for hiring decisions. Investigating associations between class membership and meaningful consequences on the job would hence be helpful to showcase the utility of such mixture models in applied measurement contexts like personnel selection.

## Conclusion

To conclude, as our simulation and empirical demonstration illustrated, the M-MNRM provides a valuable extension of latent variable models of faking. Compared to many other faking models, the M-MNRM is not restricted to a single measurement model but allows for qualitatively different response strategies employed by test-takers. Both psychometrically and from an applied measurement perspective, such an extension is worthwhile since it offers researchers and practitioners a tool to detect and account for response strategies associated with a different use of substantive traits and faking, which would otherwise bias results. Future research can help to discover boundary conditions of the model’s efficacy or alternative parameterizations that allow a sophisticated modeling of different faking tendencies.

## Supplemental Material

sj-docx-1-epm-10.1177_00131644251341843 – Supplemental material for Disentangling Qualitatively Different Faking Strategies in High-Stakes Personality Assessments: A Mixture Extension of the Multidimensional Nominal Response Model Supplemental material, sj-docx-1-epm-10.1177_00131644251341843 for Disentangling Qualitatively Different Faking Strategies in High-Stakes Personality Assessments: A Mixture Extension of the Multidimensional Nominal Response Model by Timo Seitz, Ö. Emre C. Alagöz and Thorsten Meiser in Educational and Psychological Measurement

## Notes

1. Because class membership was based on the described sampling procedure, slight deviations between the actual class proportions in a replication and the class proportions presented in the Simulation Conditions section could occur. However, deviations were very small ( M=0.000,SD=0.014,range=[−0.060,0.059] ). Also, it was made sure that classes that should be absent by design were indeed empty by using regression intercepts of −∞.

2. Due to data protection guidelines, there was no age variable in Dataset 3. According to consultations with the testing company, the approximate average age of applicants for this particular position was above 30 years, with a range from 20 to 45.

3. As is well known in the psychometric literature, mixture models can be hard to estimate in empirical data because of multimodal likelihood functions (e.g., Hipp & Bauer, 2006; McLachlan & Peel, 2000). Instead of just discarding MCMC chains that converged to a local solution, we employed a two-step estimation of the “mixture θ_S_/θ_F_ model.” The first step should optimize initial values for the actual model estimation in the second step (cf. O’Hagan et al., 2012). This was done by running 30 parallel chains in the first step (each with 2,000 iterations after a 2,000-iteration burnin phase) and using the parameter estimates from the chain that yielded the highest model likelihood as initial values for the second step. The second step then featured the described estimation with 12 parallel chains. However, to prevent that all chains started with the exact same set of initial values, we added random noise to the initial values of every chain. Details can be found at https://osf.io/vwqf3/

4. Specifically, for each MCMC iteration after the burnin phase, item responses were simulated using the model equation of the respective model and the parameters sampled in the given iteration. Then, the root of the mean squared deviation between item intercorrelations in the simulated versus observed data was computed for each iteration, before the average across iterations was calculated to yield the SRMR of a model.

5. We chose Dataset 3 for the validation analysis because item wordings in the career counseling dataset were not fully identical to item wordings in Datasets 1 and 2. Some item wordings in Datasets 1 and 2 had been adjusted to better fit the age group of applicants for the respective job (mainly high school graduates).

6. More information on the class-specific estimation of person parameters can be found in the Supplemental Material. There, we also report the debiasing effect of the mixture model on substantive trait scores in a simulation with item-category intercepts differing systematically between classes.

7. We also conducted a simulation with all covariate effects fixed to 0 in the data generation (see the Supplemental Material). Results, including the recovery of class proportions, class membership, and latent regression coefficients, were equivalent to the findings reported above. This suggests that the M-MNRM does not require class membership predictors of considerable effect size to produce satisfactory results.

8. Note that it only makes sense to interpret and rank substantive trait score estimates of test-takers from classes where item responses are influenced by substantive traits (i.e., “S&F class” and “S-only class”) As described in the Supplemental Material, the M-MNRM formally estimates substantive trait scores for test-takers in the “F-only class” (which should be close to 0), but these estimates are meaningless and should not be interpreted.
