# Supplementary material for: Disentangling Qualitatively Different Faking Strategies in High-Stakes Personality Assessments: A Mixture Extension of the Multidimensional Nominal Response Model
Source: Educ Psychol Meas. 2025 Jul 29;85(6):1237–77. doi: 10.1177/00131644251341843 (PMC12310618; doi:10.1177/00131644251341843)
Supplement: sj-docx-1-epm-10.1177_00131644251341843 – Supplemental material for Disentangling Qualitatively Different Faking Strategies in High-Stakes Personality Assessments: A Mixture Extension of the Multidimensional Nominal Response Model [file sj-docx-1-epm-10.1177_00131644251341843.docx]

# Online Supplement

## Meaning of Person Parameters of 0

As noted in the Main Text, the mixture multidimensional nominal response model (M-MNRM) does not imply that test-takers in the “S-only class” per se do not have a person parameter on the faking dimension or, correspondingly, that test-takers in the “F-only class” per se do not have person parameters on substantive trait dimensions. Instead, test-takers in the “S-only class” and “F-only class” are assumed to not consider the respective dimension(s) when responding to the items. A consequence of this is that the data contain no information on faking and substantive trait levels for test-takers in the “S-only class” and “F-only class”, respectively. A proper recovery of the respective person parameters is hence precluded in conditions in which the whole sample belongs to the “S-only class” or “F-only class” (see Figure 1 in the Main Text).

Similarly, the M-MNRM is not equivalent to a non-mixture MNRM in which “S-only” test-takers (“F-only” test-takers) have faking person parameters (substantive trait person parameters) of 0. A person parameter of 0 simply reflects one possible value on the latent continuum of the dimension, which is oftentimes defined as the latent mean. If one was to identify the scale of person parameters by setting the latent mean to any other real value, response patterns previously leading to a person parameter of 0 would then lead to a different person parameter value, because a change in the latent mean would be accompanied by a change in item-category intercepts. It is hence crucial to note that a person parameter of 0 does not imply the absence of a given dimension. Instead, the absence of a dimension is reflected in an item slope of 0, which implies that a dimension explains no variance in item responses. Item slopes of 0 hence capture the idea of qualitatively different response strategies characterized by a different set of dimensions influencing item responding (see also Alagöz & Meiser, 2024). Person parameters of 0, on the contrary, are just one quantitative score on a dimension.

To illustrate the non-equivalence of the mixture and non-mixture MNRM, we reanalyzed the first replication of the first condition (all classes equally sized) from the simulation reported in the Main Text. If the M-MNRM (“mixture θ_S_ / θ_F_ model”) was equivalent to its non-mixture version (“θ_S_ / θ_F_ model”), the “θ_S_ / θ_F_ model” should estimate faking person parameters of “S-only class” test-takers and substantive trait person parameters of “F-only class” test-takers (close) to 0. However, as can be seen in Figure S1, “S-only class” test-takers had estimated faking person parameters that were on average smaller than 0 with non-zero variation ($M_{F}=-0.30$, $SD_{F}=0.22$). Correspondingly, “F-only class” test-takers had on average non-zero substantive trait person parameter estimates with considerable variation ($M_{S1}=-0.45$, ${SD}_{S1}=0.43$; $M_{S2}=-0.13$, ${SD}_{S2}=0.43$; $M_{S3}=-0.27$, ${SD}_{S3}=0.39$). These results could also be found in other replications and simulation conditions. Other than that, the consistent selection of the “mixture θ_S_ / θ_F_ model” over the “θ_S_ / θ_F_ model” in simulation conditions with more than one class in the data (see Simulation Results section in the Main Text) provides additional evidence for the importance of accounting for multiple latent classes in the model.

**Figure S1**

*Class-Specific Distributions of Estimated Person Parameters in the “θ_S_ / θ_F_ Model”*


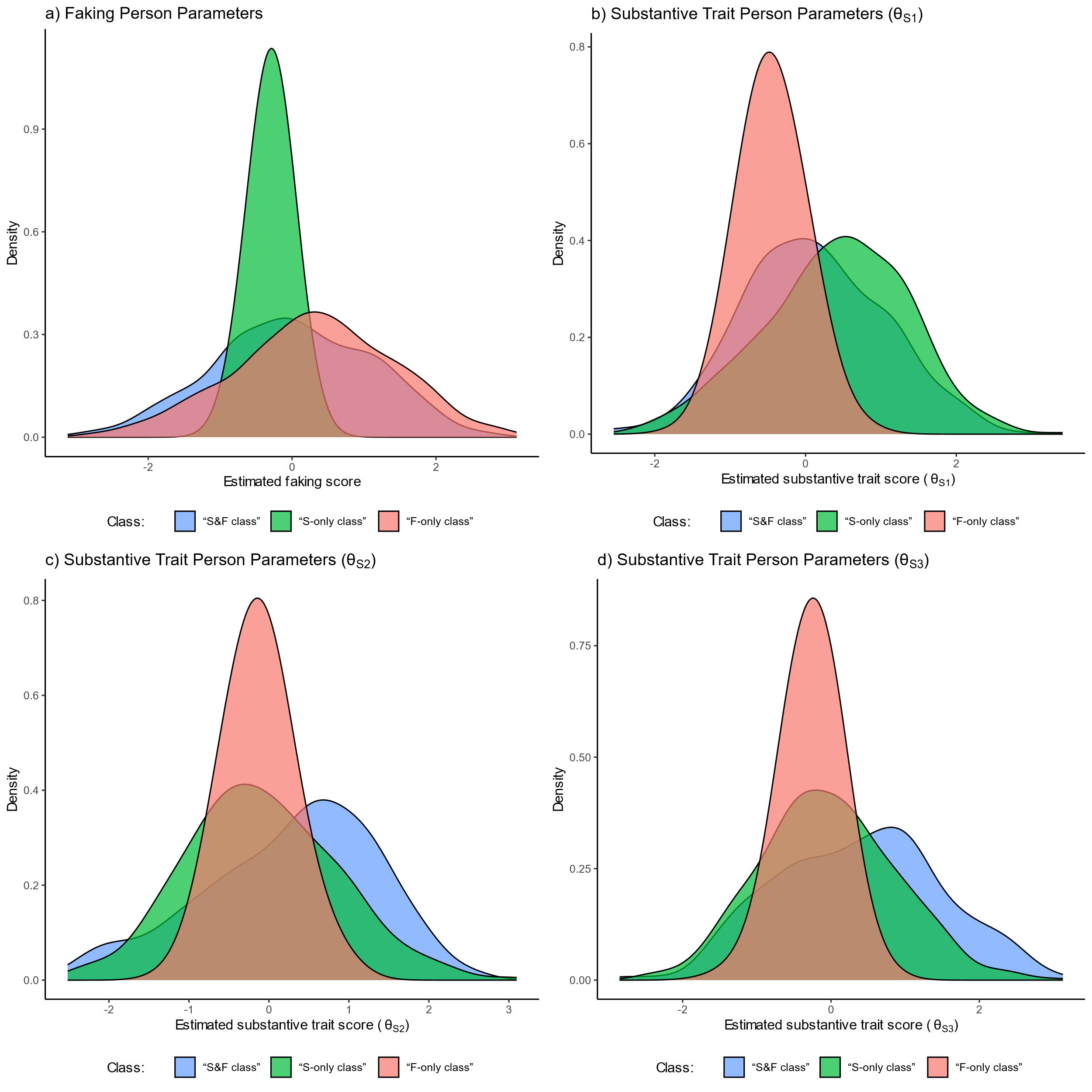


*Note.* Exemplary illustration for the first replication of the simulation condition with equal class proportions. The pattern for other replications and conditions was analogous. Plots display kernel densities of estimated faking (Panel a) or substantive trait (Panels b to d) scores in the non-mixture multidimensional nominal response model (MNRM; “θ_S_ / θ_F_ model”).

## Information on the Class-Specific Estimation of Person Parameters

In the non-mixture MNRM of faking, the estimation of person parameters is based on a single measurement model, namely on a measurement model in which substantive traits as well as faking influence item responses. Thus, all test-takers receive substantive trait score estimates that are adjusted for their estimated faking score, which itself is estimated whilst taking substantive trait levels into account. However, in the M-MNRM, the estimation of person parameters is based on the measurement model of the class a test-taker is assigned to. That is, only test-takers for whom the model implies that responses have been influenced by substantive traits *and* faking (i.e., test-takers assigned to the “S&F class”) receive substantive trait score estimates adjusted for faking.^[[1]](#footnote-2)^ Test-takers for whom the model implies that responses have not been influenced by faking (i.e., test-takers assigned to the “S-only class”) receive substantive trait score estimates that are not adjusted for faking. Test-takers for whom the model implies that responses have only been influenced by faking (i.e., test-takers assigned to the “F-only class”) technically also receive substantive trait score estimates, which will be close to 0 because the Markov chain Monte Carlo (MCMC) algorithm for these test-takers just samples from the prior distributions of person parameters that are centered around 0 in our case. Crucially, given the “F-only” classification, one would in any case not interpret such estimated values, nor report substantive trait scores for the respective test-takers in applied assessment contexts.^[[2]](#footnote-3)^

### Simulation With Systematically Different Class-Specific Item-Category Intercepts

To illustrate the debiasing effect of the M-MNRM on substantive trait score estimates, we ran a simulation that differed from the one reported in the Main Text only in the fact that the true item-category intercepts were systematically different between classes. To generate such intercept parameters, we proceeded as follows:

- For the “S-only class”, intercepts (via cumulative thresholds) were generated like in the main simulation.
- For the “S&F class”, the thresholds of the “S-only class” were taken as a basis, but were shifted according to the items’ desirability characteristics. This was done by subtracting a value drawn from $N(1, {0.2}^{2})$ from each non-fixed category threshold if an item had a monotonically increasing desirability trajectory, whereas a value from $N(0.5, {0.2}^{2})$ was subtracted if an item had a nonmonotonically increasing desirability trajectory and a value from $N(0, {0.2}^{2})$ was subtracted if an item had an inverted-U-shaped desirability trajectory. The shifted thresholds were then transformed into intercepts.
- For the “F-only class”, intercepts were generated like in the main simulation, however, the center of the distribution for sampling category thresholds was shifted according to the items’ desirability characteristics. If an item had a monotonically increasing desirability trajectory, the center was shifted negatively by a value drawn from $N(1.5, {0.2}^{2})$. If an item had a nonmonotonically increasing desirability trajectory, the magnitude of the negative shift was drawn from $N(0.75, {0.2}^{2})$, whereas the magnitude of the shift was drawn from $N(0, {0.2}^{2})$ if an item had an inverted-U-shaped desirability trajectory. The sampled thresholds were then again transformed into intercepts.

This procedure should create a data situation that is empirically more realistic: First, intercepts in the “S-only class” and “S&F class” were not independent, which makes sense considering that substantive traits influence item responses in both classes. Second, the shift of thresholds and intercepts in the “S&F class” and “F-only class” meant that item response distributions differed systemically between classes. Within the latter two classes, items with a monotonically increasing desirability trajectory now had higher means than items with a nonmonotonically increasing desirability trajectory, which themselves had higher means than items with an inverted-U-shaped desirability trajectory. Because of the different magnitudes of threshold shifts, differences in item means were more pronounced in the “F-only class” than in the “S&F class”. Even though means of items with an inverted-U-shaped desirability trajectory did not differ systematically between the three classes, the described data generation had the consequence that simulated item responses were on average highest in the “F-only class” and lowest in the “S-only class”. This shift of response distributions is in line with research showing that faking biases responses into the direction of social desirability (Birkeland et al., 2006; Hu & Connelly, 2021; Viswesvaran & Ones, 1999).

Figure S2 depicts scatterplots of estimated and true substantive trait scores in the “θ_S_ model”, “θ_S_ / θ_F_ model”, and “mixture θ_S_ / θ_F_ model” (exemplarily for the representative case of the first substantive trait in the first replication of the condition with equal class proportions). Unsurprisingly, the mean differences in generated item responses between classes translated into mean differences in substantive trait score estimates in the “θ_S_ model”, which represented a regular multidimensional item response theory (IRT) model for ordinal data. In the “θ_S_ / θ_F_ model”, in which faking was accounted for as an additional latent dimension, substantive trait score estimates were more closely associated with their true values. However, there were still considerable mean differences between classes. Only in the “mixture θ_S_ / θ_F_ model”, which included the three latent classes related to different response strategies, mean differences were eliminated. Thus, if a non-mixture model (or sum scores) were used, “F-only” test-takers would on average receive the highest assessment scores, which would put them at an unjustified advantage for high-stakes decisions like in personnel selection. “S-only” test-takers, in contrast, would on average receive the lowest assessment scores, such that their chances of being selected for a job, promotion, or the like would be disproportionately reduced. If, however, the M-MNRM was used, “S-only” and “S&F” test-takers would on average get the same scores, whereas “F-only” test-takers would have substantive trait score estimates close to the prior mean (0 in our case). Since responses of “F-only” test-takers contain no information on their substantive trait levels, it would not be sensible to report any scores for this group. Instead, one could recommend making use of other assessment tools for these test-takers, such as interviews or more faking-resistant measures of personality.

Apart from the debiasing effect on substantive trait score estimates, the results of the simulation with systematically different class-specific item-category intercepts were very similar to the results of the simulation reported in the Main Text, especially regarding the recovery of item parameters, latent correlations, and latent regression coefficients. A noteworthy difference were hit rates, which were generally lower in the simulation with systematically different intercepts than in the simulation reported in the Main Text (which featured *un*systematically different intercepts). As can be seen in Table S1, this mainly applied to the hit rates in the “S&F class” and the “S-only class”. The reduced classification accuracy in these classes suggests that having non-independent class-specific intercepts (which was the case for the “S&F class” and “S-only class”) can hamper class separability, as there is less unique information in the data that can be used for properly assigning test-takers to classes. Considering that class-specific intercepts could well be non-independent in empirical situations, the hit rates in this additional simulation might in fact be more realistic for the M-MNRM’s classification accuracy in empirical data than the hit rates in the main simulation (cf. Table 2 in the Main Text). Nonetheless, even in the case of systematically different class-specific intercepts, hit rates were still high in an absolute sense (all above 90%). Also, class proportions were recovered without bias and with comparable precision as in the main simulation, and the mean entropy value across replications and conditions was only slightly smaller than in the main simulation (.972 vs. .992).

**Figure S2**

*Scatterplots of Estimated and True Substantive Trait Scores in Different Models*


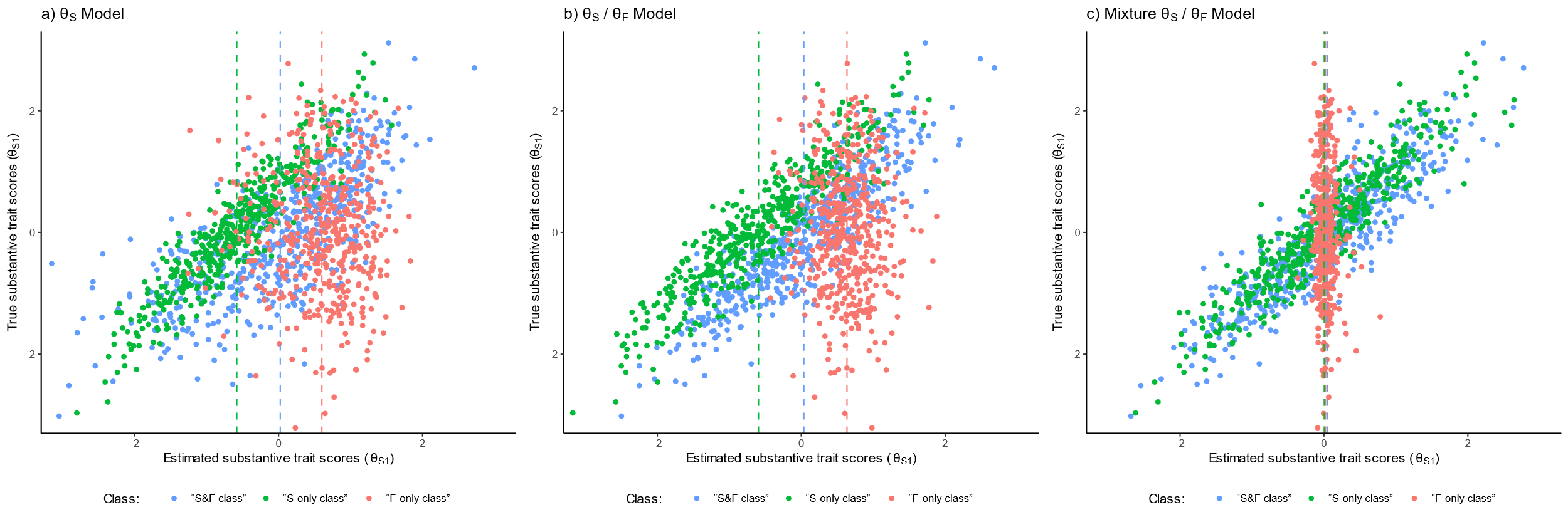


*Note.* Exemplary illustration for the first substantive trait in the first replication of the simulation condition with equal class proportions (simulation with systematically different class-specific item-category intercepts). The pattern for the other substantive traits as well as replications and conditions was analogous. Dashed vertical lines represent the class-specific mean substantive trait score estimate.

**Table S1**

*Recovery of Class Proportions and Hit Rates in the Simulation With Systematically Different Class-Specific Item-Category Intercepts*

|  | Class | | | | | | | |
| --- | --- | --- | --- | --- | --- | --- | --- | --- |
|  | “S&F class” | |  | “S-only class” | |  | “F-only class” | |
| Condition | Bias  (RMSE) | HR |  | Bias  (RMSE) | HR |  | Bias  (RMSE) | HR |
| Classes equally sized | .004  (.009) | .945 |  | –.004  (.009) | .950 |  | .000  (.003) | .972 |
| “S&F class” dominant | .004  (.009) | .968 |  | –.003  (.007) | .921 |  | –.001  (.003) | .968 |
| “S-only class” dominant | –.006  (.012) | .907 |  | .005  (.011) | .976 |  | .000  (.002) | .977 |
| “F-only class” dominant | –.003  (.008) | .930 |  | .002  (.007) | .957 |  | .000  (.002) | .982 |
| “S&F class” absent | .003  (.005) |  |  | –.001  (.003) | .985 |  | –.001  (.002) | .985 |
| “S-only class” absent | –.003  (.005) | .977 |  | .002  (.004) |  |  | .000  (.002) | .980 |
| “F-only class” absent | –.006  (.013) | .951 |  | .004  (.010) | .961 |  | .002  (.004) |  |
| only “S&F class” present | –.004  (.009) | .985 |  | .002  (.004) |  |  | .002  (.005) |  |
| only “S-only class” present | .002  (.004) |  |  | –.003  (.006) | .985 |  | .001  (.003) |  |
| only “F-only class” present | .002  (.003) |  |  | .001  (.003) |  |  | –.003  (.006) | .984 |

*Note.* Values reflect the mean bias and root mean square error (RMSE; in brackets) of estimated class proportions across replications within a condition, as well as hit rates (HR). Hit rates are the mean percentages of simulated test-takers correctly assigned to their respective class.

## Simulation With Only Null-Effect Covariates

We also ran the simulation reported in the Main Text with only null-effect covariates. This had the purpose of investigating whether the M-MNRM can also yield satisfactory results if class membership cannot be predicted by covariates. In this additional simulation, we generated the data using the same procedure as in the main simulation, but with all latent regression slopes fixed to 0 (latent regression intercepts were still specified to produce the class proportions of the respective simulation condition). We restricted this simulation to 5 replications per condition.

As can be seen in the following tables and figures, results were essentially the same as in the main simulation that featured null-, weak-, and strong-effects predictors of class membership. It would have been a reasonable assumption that the M-MNRM requires covariate effects of considerable size to inform the parameters specific to mixture models, such as class proportions, class membership, and class-specific item parameters. However, the results of this additional simulation suggest that the M-MNRM also works fine if there are no covariates that significantly predict class membership.

**Table S2**

*Percentages of Correctly Selected Models in the Simulation With Only Null-Effect Covariates*

|  | Model selection criterion | | |
| --- | --- | --- | --- |
| Condition | DIC | WAIC | LOOIC |
| Classes equally sized | 100.0%  (100.0%) | 100.0%  (100.0%) | 100.0%  (100.0%) |
| “S&F class” dominant | 100.0%  (100.0%) | 100.0%  (100.0%) | 100.0%  (100.0%) |
| “S-only class” dominant | 100.0%  (100.0%) | 100.0%  (100.0%) | 100.0%  (100.0%) |
| “F-only class” dominant | 100.0%  (100.0%) | 100.0%  (100.0%) | 100.0%  (100.0%) |
| “S&F class” absent | 100.0%  (100.0%) | 100.0%  (100.0%) | 100.0%  (100.0%) |
| “S-only class” absent | 100.0%  (100.0%) | 100.0%  (100.0%) | 100.0%  (100.0%) |
| “F-only class” absent | 100.0%  (100.0%) | 100.0%  (100.0%) | 100.0%  (100.0%) |
| only “S&F class” present | 80.0%  (100.0%) | 100.0%  (100.0%) | 80.0%  (100.0%) |
| only “S-only class” present | 60.0%  (80.0%) | 80.0%  (100.0%) | 80.0%  (100.0%) |
| only “F-only class” present | 80.0%  (100.0%) | 80.0%  (100.0%) | 80.0%  (100.0%) |

*Note.* Percentages are based on 5 replications per condition. In simulation conditions in which more than one class was present, the “mixture θ_S_ / θ_F_ model” was the underlying population model, whereas either the “θ_S_ / θ_F_ model”, “θ_S_ model”, or “θ_F_ model” was the population model in conditions in which only the respective class was present. Values in brackets reflect percentages of correct decisions concerning the question of whether a mixture or non-mixture model was the data-generating model. DIC = deviance information criterion; WAIC = widely applicable information criterion; LOOIC = leave-one-out information criterion.

**Table S3**

*Recovery of Class Proportions and Hit Rates in the Simulation With Only Null-Effect Covariates*

|  | Class | | | | | | | |
| --- | --- | --- | --- | --- | --- | --- | --- | --- |
|  | “S&F class” | |  | “S-only class” | |  | “F-only class” | |
| Condition | Bias  (RMSE) | HR |  | Bias  (RMSE) | HR |  | Bias  (RMSE) | HR |
| Classes equally sized | .000  (.003) | .969 |  | .000  (.001) | .978 |  | .000  (.003) | .976 |
| “S&F class” dominant | .003  (.006) | .978 |  | –.002  (.006) | .963 |  | –.001  (.004) | .969 |
| “S-only class” dominant | –.001  (.004) | .970 |  | .001  (.002) | .983 |  | .000  (.003) | .981 |
| “F-only class” dominant | –.001  (.004) | .971 |  | .001  (.003) | .982 |  | .000  (.003) | .982 |
| “S&F class” absent | .003  (.005) |  |  | –.001  (.003) | .984 |  | –.001  (.003) | .984 |
| “S-only class” absent | –.003  (.007) | .978 |  | .002  (.004) |  |  | .001  (.002) | .981 |
| “F-only class” absent | –.003  (.006) | .978 |  | .001  (.002) | .979 |  | .002  (.004) |  |
| only “S&F class” present | –.004  (.009) | .985 |  | .002  (.004) |  |  | .002  (.005) |  |
| only “S-only class” present | .002  (.003) |  |  | –.003  (.006) | .985 |  | .001  (.003) |  |
| only “F-only class” present | .002  (.003) |  |  | .002  (.003) |  |  | –.003  (.006) | .985 |

*Note.* Values reflect the mean bias and root mean square error (RMSE; in brackets) of estimated class proportions across replications within a condition, as well as hit rates (HR). Hit rates are the mean percentages of simulated test-takers correctly assigned to their respective class.

**Table S4**

*Recovery of Latent Regression Coefficients in the Simulation With Only Null-Effect Covariates*

|  | Bias (RMSE) of regression coefficients | |
| --- | --- | --- |
| Condition | Intercepts | Slopes |
| Classes equally sized | 0.00  (0.07) | 0.00  (0.06) |
| “S&F class” dominant | –0.03  (0.07) | 0.01  (0.05) |
| “S-only class” dominant | –0.01  (0.04) | –0.01  (0.08) |
| “F-only class” dominant | 0.00  (0.06) | –0.03  (0.07) |
| “S&F class” absent | 0.01  (0.05) | 0.01  (0.04) |
| “S-only class” absent | –0.01  (0.05) | 0.00  (0.05) |
| “F-only class” absent | 0.02  (0.04) | –0.01  (0.04) |

*Note.* Values reflect the mean bias and root mean square error (RMSE; in brackets) of estimated latent regression coefficients across replications within a condition. Results for slopes are aggregated across the three null-effect covariates used in this simulation. In the condition in which the “S&F class” was absent, the “S-only class” is treated as the reference class. Results for conditions with only one class in the data are left out because class membership is a constant in these conditions, which precludes a proper recovery of regression coefficients.

**Figure S3**

*Recovery of Substantive Trait and Faking Scores in the Simulation With Only Null-Effect Covariates*


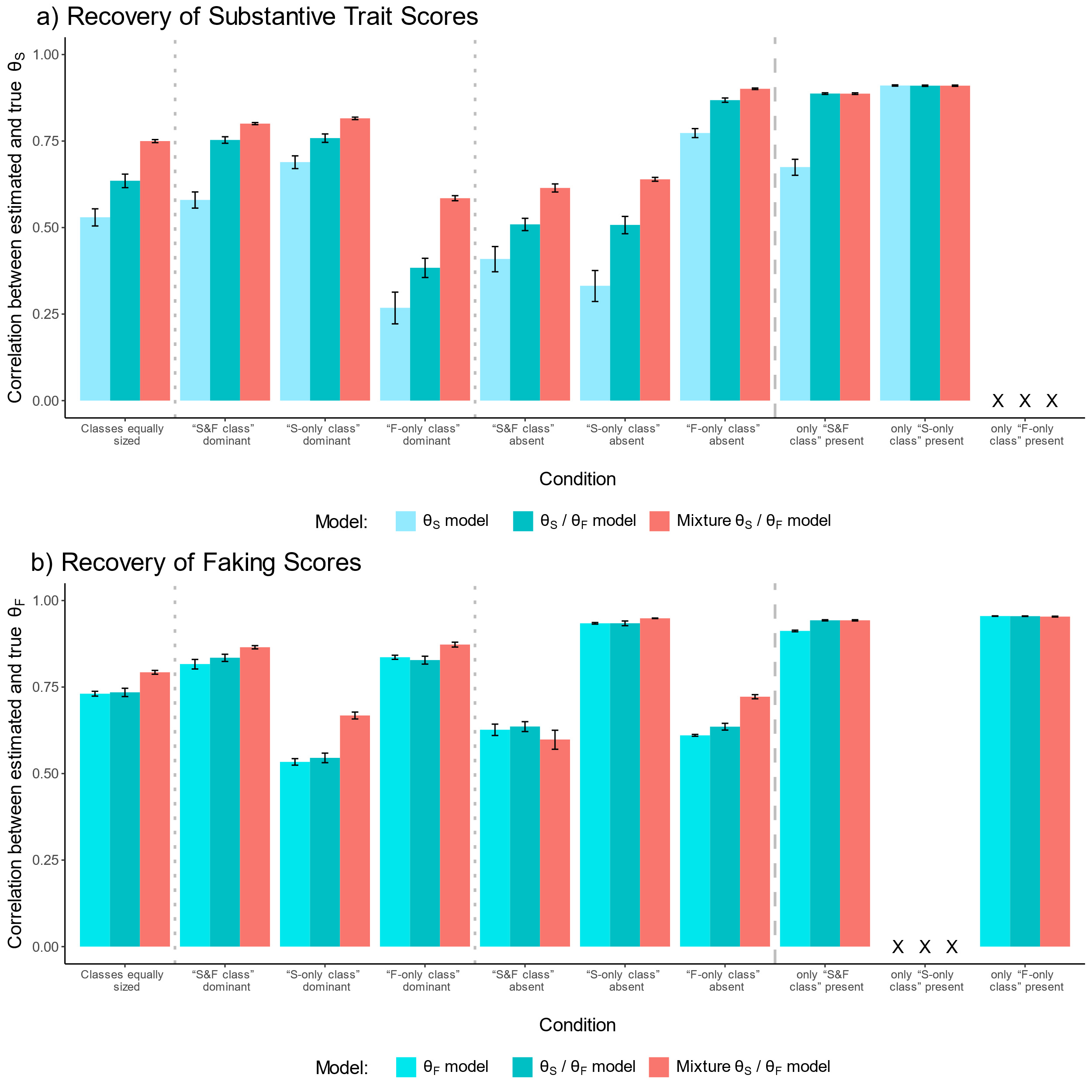


*Note.* Values reflect the mean correlations (using Fisher’s *z*-transformation) between estimated and true substantive trait scores (Panel a) or faking scores (Panel b) across replications within a condition. Results for substantive traits are aggregated across the three substantive traits used in the simulation. Error bars represent the standard error of the mean. “X” denotes that a proper recovery of the particular parameters is precluded in the respective condition because they have not influenced item responses in the data generation.

**Figure S4**

*Recovery of Item Slopes in the Simulation With Only Null-Effect Covariates*


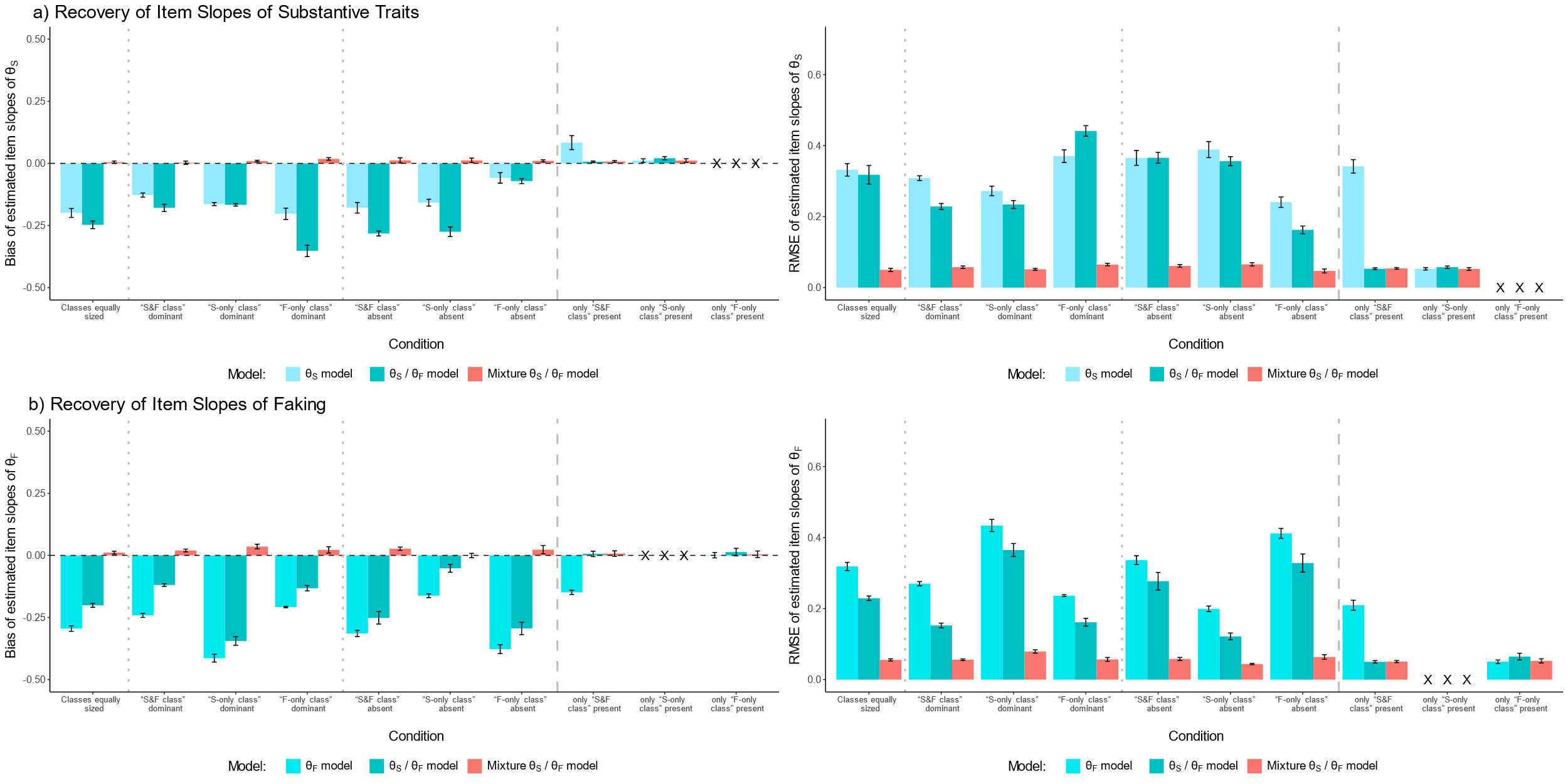


*Note.* Values reflect the mean bias or root mean square error (RMSE) of estimated item slopes of substantive traits (Panel a) or faking (Panel b) across replications within a condition. Error bars represent the standard error of the mean. “X” denotes that a proper recovery of the particular parameters is precluded in the respective condition because they have not influenced item responses in the data generation.

**Figure S5**

*Recovery of Item-Category Intercepts in the Simulation With Only Null-Effect Covariates*


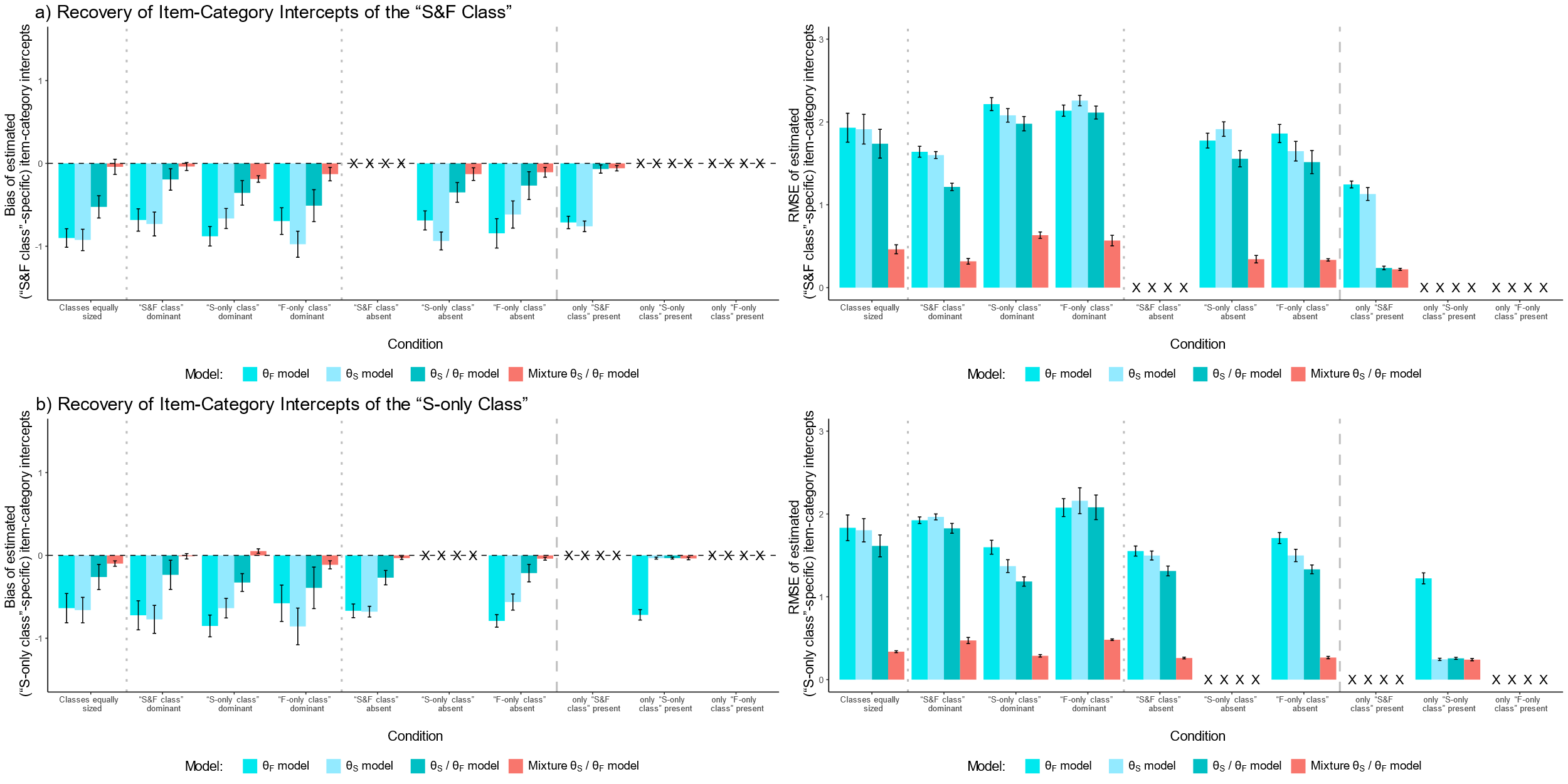


*(Continued on next page)*


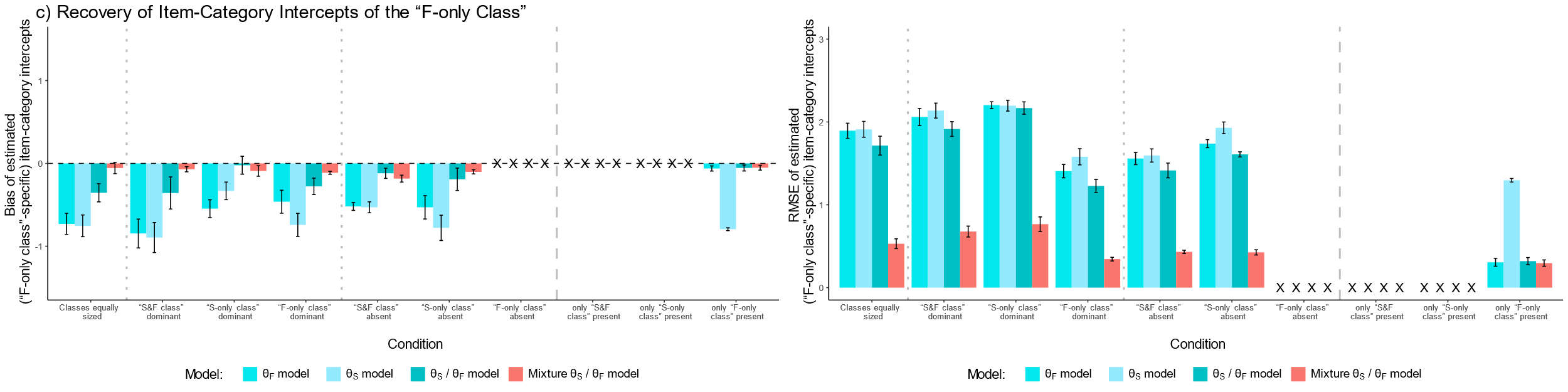


*Note.* Values reflect the mean bias or root mean square error (RMSE) of estimated item-category intercepts with respect to the true values in the “S&F class” (Panel a), “S-only class” (Panel b), or “F-only class” (Panel c) across replications within a condition. For the mixture model, the respective class-specific intercept estimates are considered. Error bars represent the standard error of the mean. “X” denotes that a proper recovery of the particular parameters is precluded in the respective condition because they have not influenced item responses in the data generation.

**Figure S6**

*Recovery of Latent Correlations in the Simulation With Only Null-Effect Covariates*


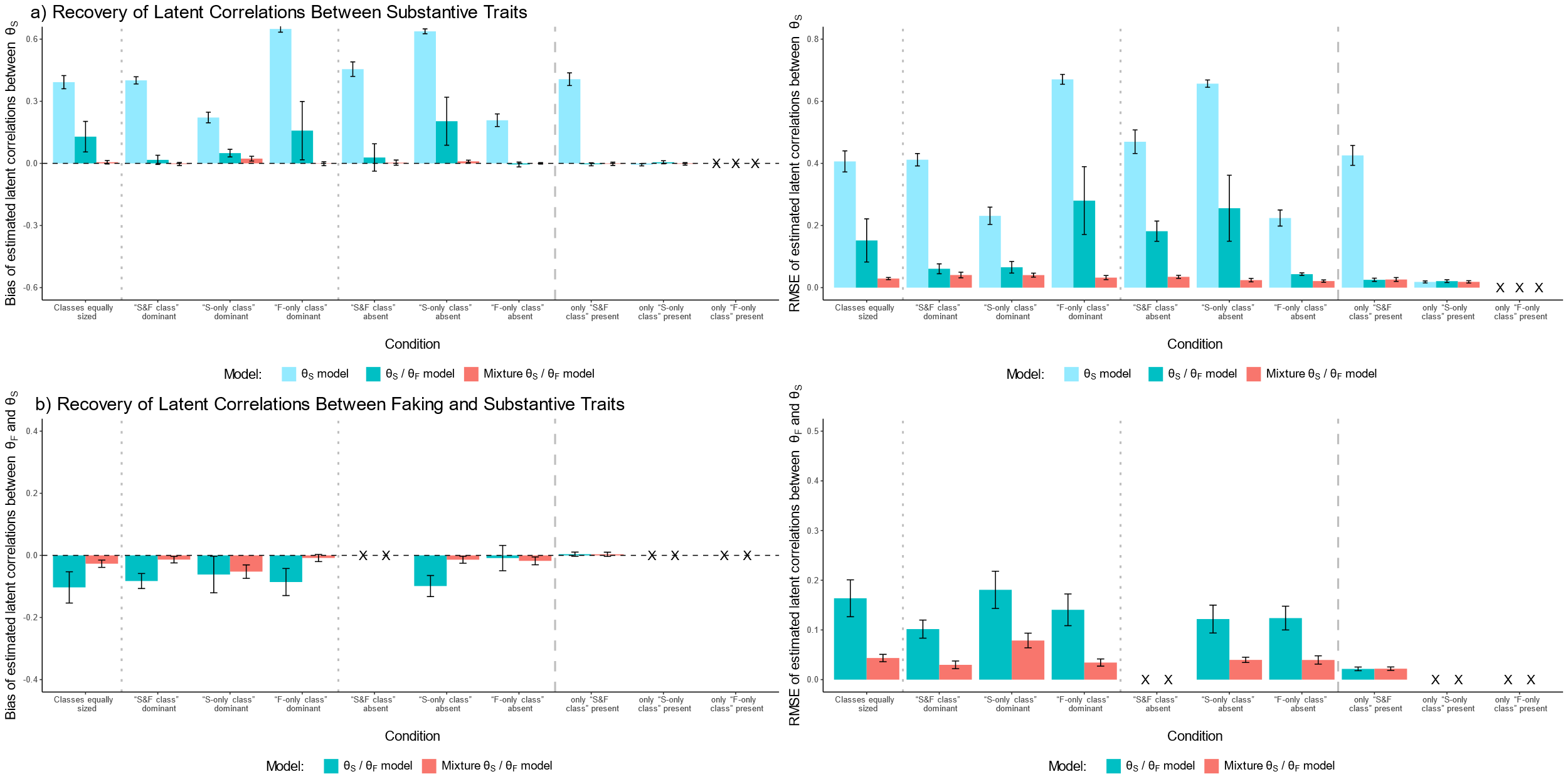


*Note.* Values reflect the mean bias or root mean square error (RMSE) of estimated latent correlations between substantive traits (Panel a) or faking and substantive traits (Panel b) across replications within a condition. Results are aggregated across the three substantive traits used in the simulation. Error bars represent the standard error of the mean. “X” denotes that a proper recovery of the particular parameters is precluded in the respective condition because they have not influenced item responses in the data generation.

## Simulation With Non-Equidistant Scoring Weights of Faking

In the main simulation, scoring weights of faking were equidistant within the segments of the desirability trajectories (see Figure 1 in Seitz et al., 2024). However, the relation between response categories and desirability may well take on idiosyncratic forms in empirical settings (Kuncel & Tellegen, 2009). To check the sensitivity of the M-MNRM to situations in which categories are related to desirability in idiosyncratic, non-equidistant ways, we ran an additional simulation. In this simulation, we proceeded as in the main simulation but used different scoring weights of faking. Specifically, we set scoring weights of faking to values collected in the pilot study for Dataset 1 (application for a bank apprenticeship) of our empirical demonstration. These scoring weights featured relations between categories and desirability that were highly item-specific and showed indeed idiosyncratic desirability trajectories (see Table S8). We ran 5 replications in this simulation and focused on the condition with equal class proportions.

The following tables and figures show essentially the same result pattern as in the main simulation, with the M-MNRM still yielding a good recovery of class proportions, class membership, and latent regression coefficients, while outperforming non-mixture models in the recovery of person and item parameters as well as latent correlations. This provides first evidence the M-MNRM is not highly sensitive to deviations from equidistant scoring weights of faking. However, a more fine-grained study would be welcome to examine the role of item desirability characteristics in the M-MNRM in more detail (cf. Seitz et al., 2024).

**Table S5**

*Percentages of Correctly Selected Models in the Simulation With Non-Equidistant Scoring Weights of Faking*

| Model selection criterion | | |
| --- | --- | --- |
| DIC | WAIC | LOOIC |
| 100.0%  (100.0%) | 100.0%  (100.0%) | 100.0%  (100.0%) |

*Note.* Percentages are based on 5 replications per condition. The “mixture θ_S_ / θ_F_ model” was the underlying population model. Values in brackets reflect percentages of correct decisions concerning the question of whether a mixture or non-mixture model was the data-generating model. DIC = deviance information criterion; WAIC = widely applicable information criterion; LOOIC = leave-one-out information criterion.

**Table S6**

*Recovery of Class Proportions and Hit Rates in the Simulation With Non-Equidistant Scoring Weights of Faking*

| Class | | | | | | | |
| --- | --- | --- | --- | --- | --- | --- | --- |
| “S&F class” | |  | “S-only class” | |  | “F-only class” | |
| Bias  (RMSE) | HR |  | Bias  (RMSE) | HR |  | Bias  (RMSE) | HR |
| .000  (.001) | .981 |  | .000  (.001) | .982 |  | .000  (.001) | .983 |

*Note.* Values reflect the mean bias and root mean square error (RMSE; in brackets) of estimated class proportions across replications, as well as hit rates (HR). Hit rates are the mean percentages of simulated test-takers correctly assigned to their respective class.

**Table S7**

*Recovery of Latent Regression Coefficients in the Simulation With Non-Equidistant Scoring Weights of Faking*

| Bias (RMSE) of regression coefficients | | | |
| --- | --- | --- | --- |
| Intercepts | Slopes of null-effect covariate | Slopes of weak-effects covariate | Slopes of strong-effects covariate |
| –0.07  (0.08) | –0.04  (0.07) | 0.01  (0.06) | 0.02  (0.13) |

*Note.* Values reflect the mean bias and root mean square error (RMSE; in brackets) of estimated latent regression coefficients across replications.

**Figure S7**

*Recovery of Substantive Trait and Faking Scores in the Simulation With Non-Equidistant Scoring Weights of Faking*

a) Recovery of Substantive Trait Scores


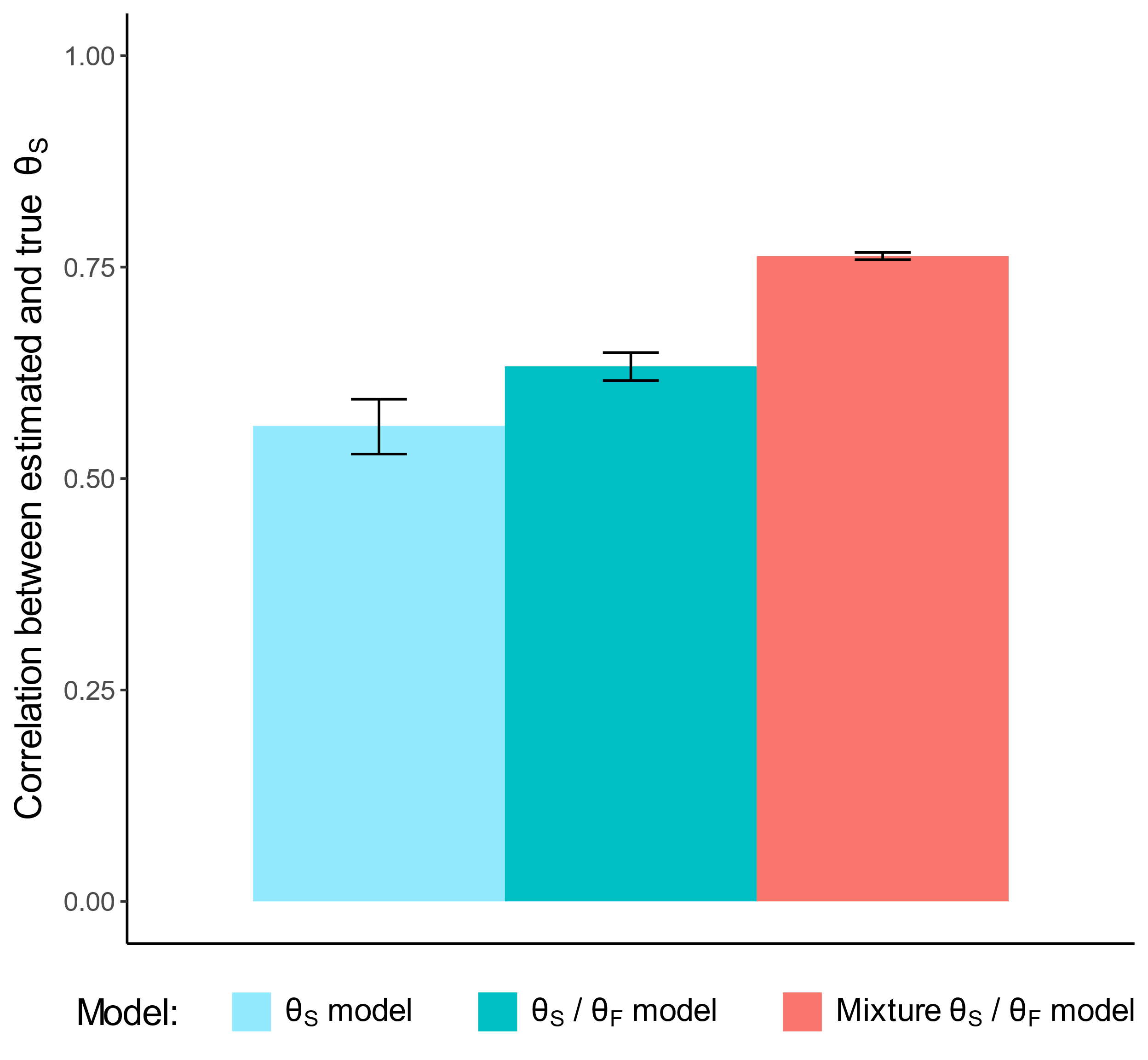


b) Recovery of Faking Scores


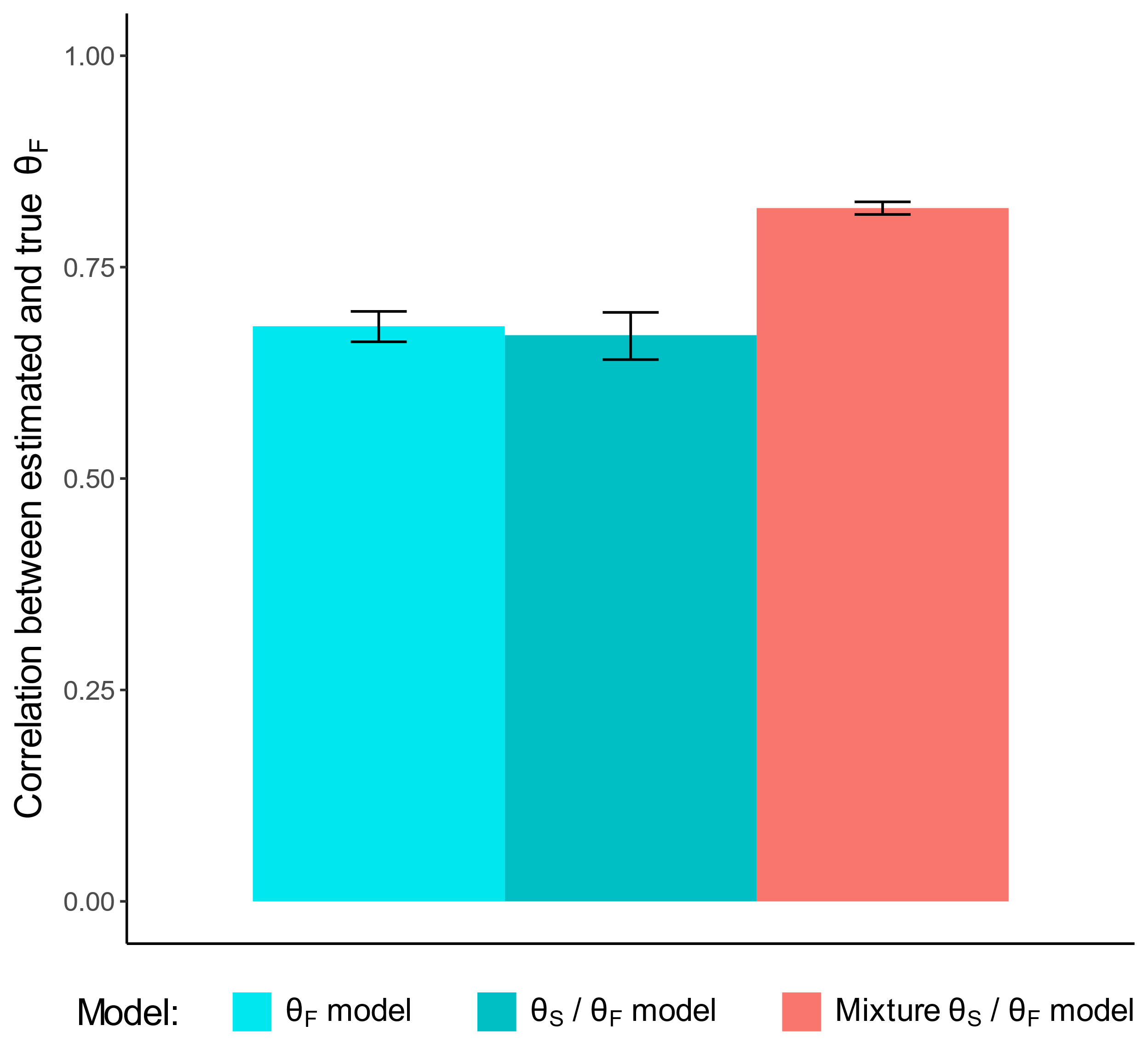


*Note.* Values reflect the mean correlations (using Fisher’s *z*-transformation) between estimated and true substantive trait scores (Panel a) or faking scores (Panel b) across replications. Results for substantive traits are aggregated across the three substantive traits used in the simulation. Error bars represent the standard error of the mean.

**Figure S8**

*Recovery of Item Slopes in the Simulation With Non-Equidistant Scoring Weights of Faking*

a) Recovery of Item Slopes of Substantive Traits


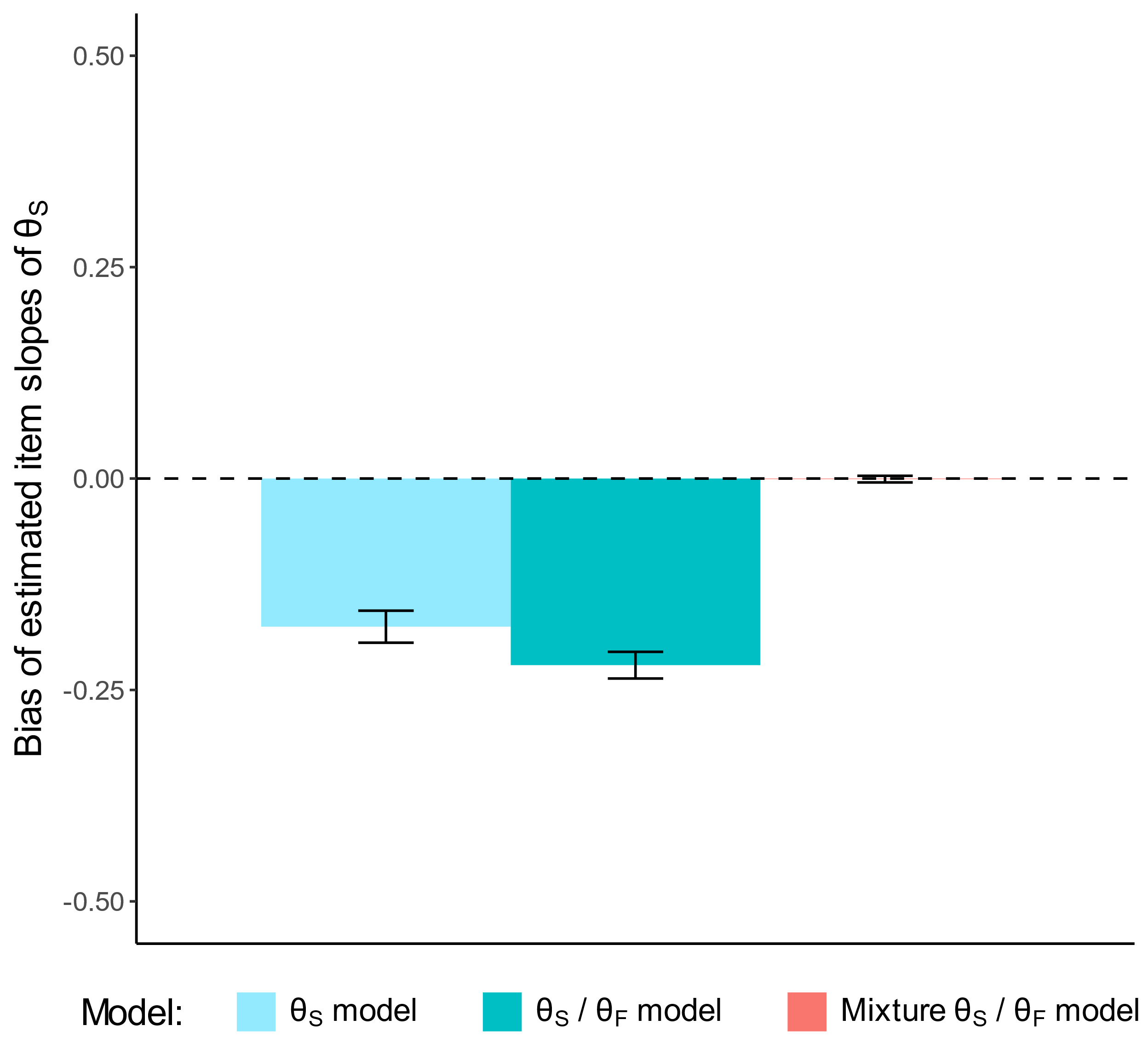

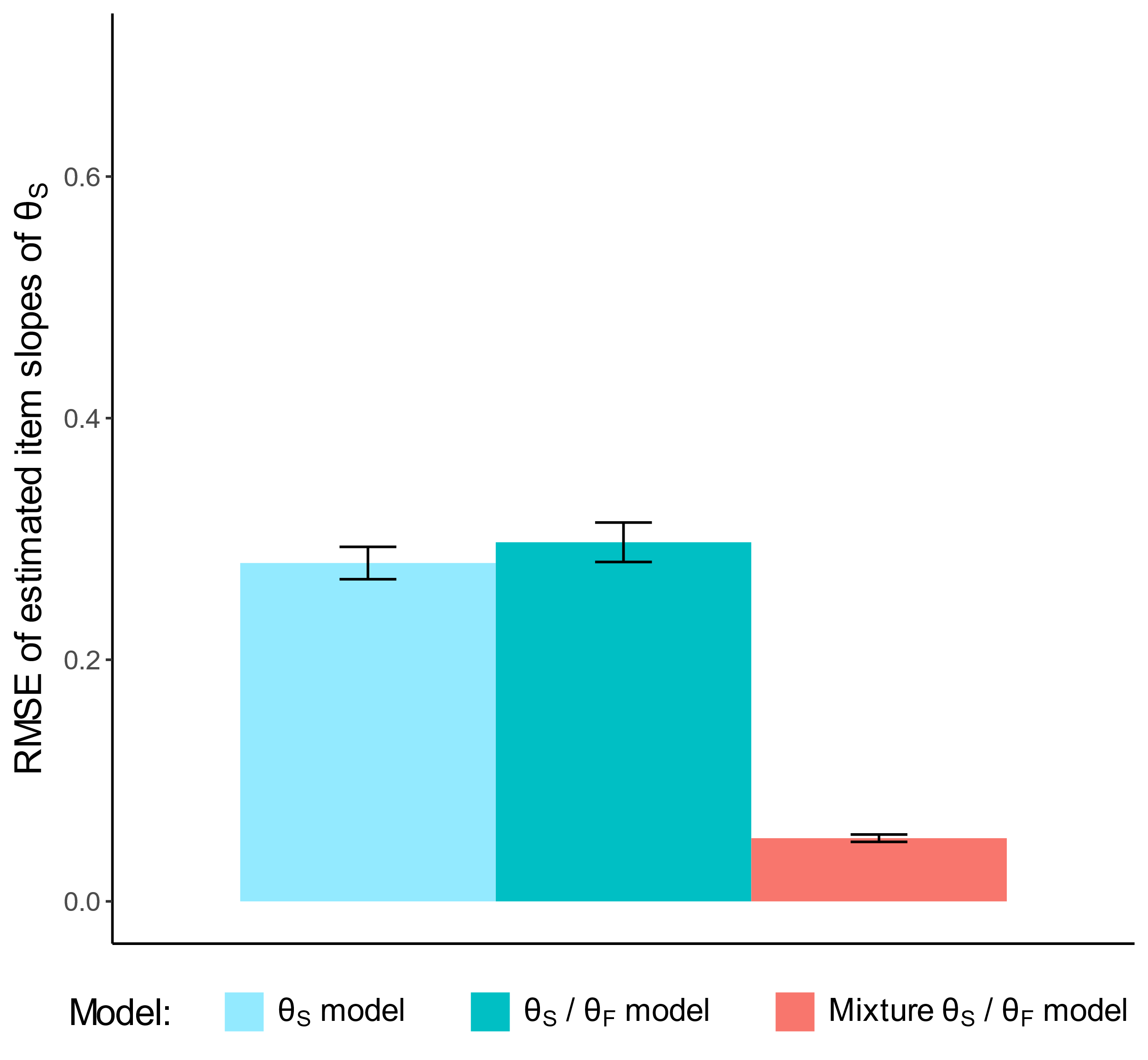


b) Recovery of Item Slopes of Faking


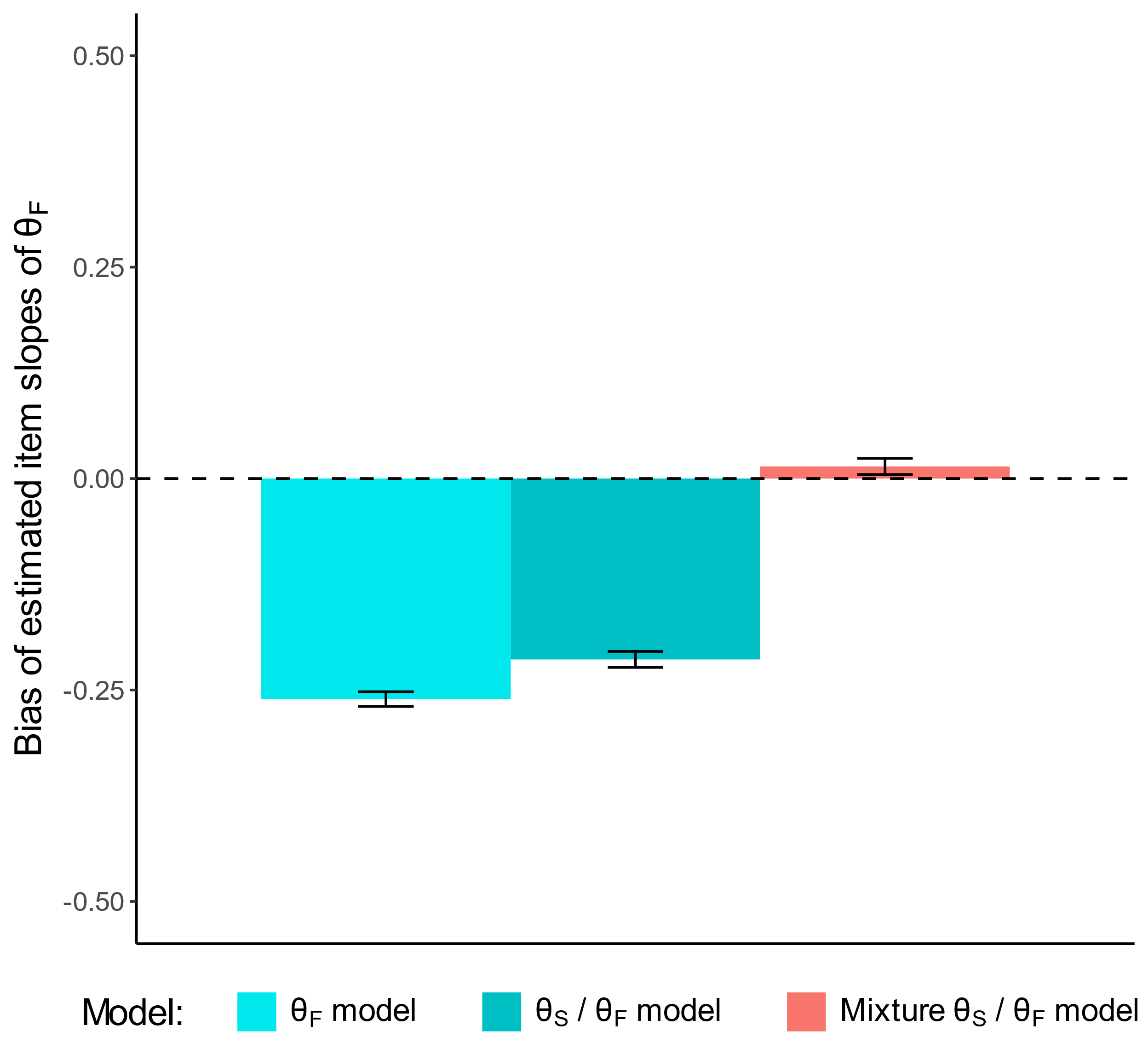

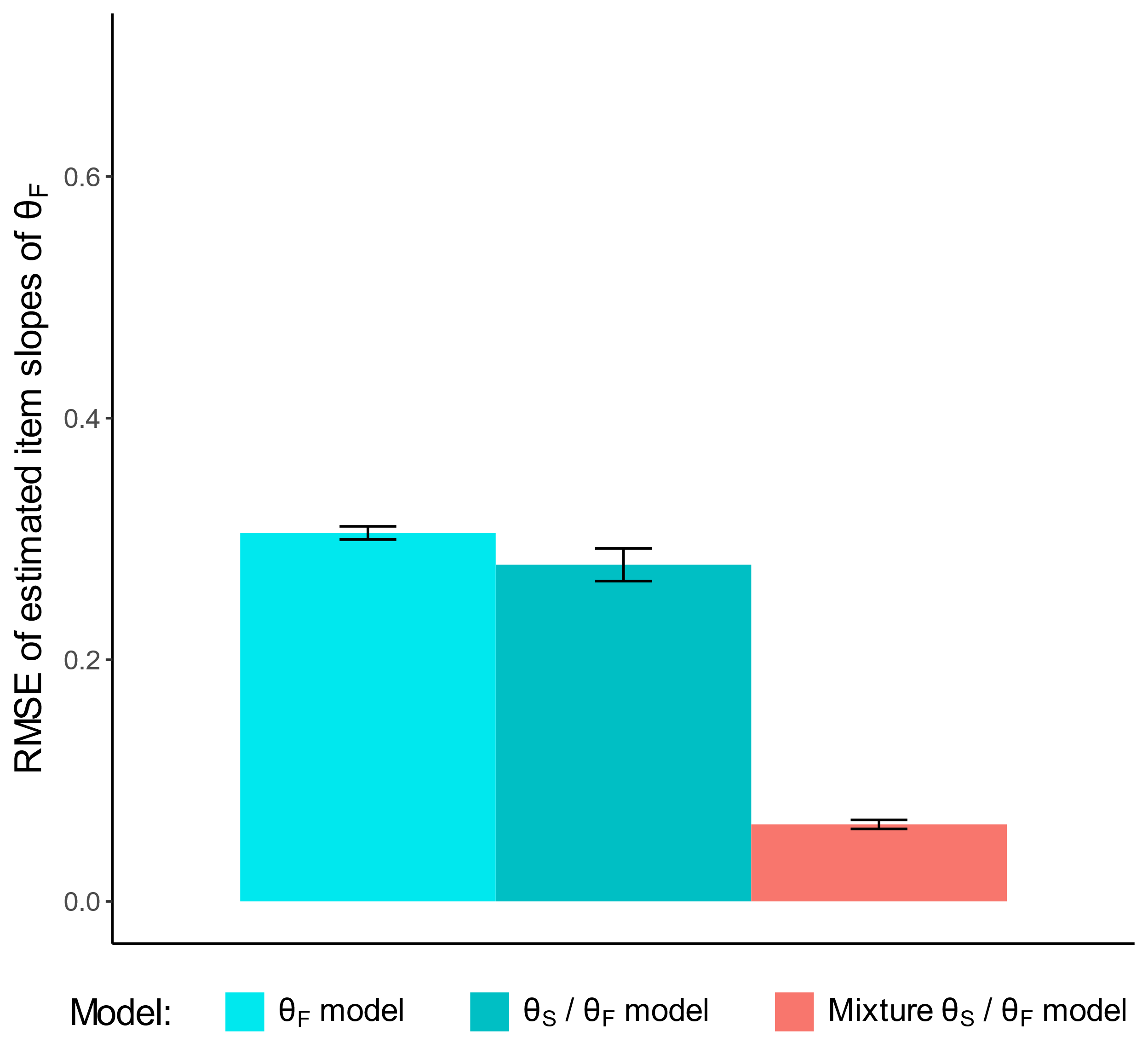


*Note.* Values reflect the mean bias or root mean square error (RMSE) of estimated item slopes of substantive traits (Panel a) or faking (Panel b) across replications. Error bars represent the standard error of the mean.

**Figure S9**

*Recovery of Item-Category Intercepts in the Simulation With Non-Equidistant Scoring Weights of Faking*

a) Recovery of Item-Category Intercepts of the “S&F Class”


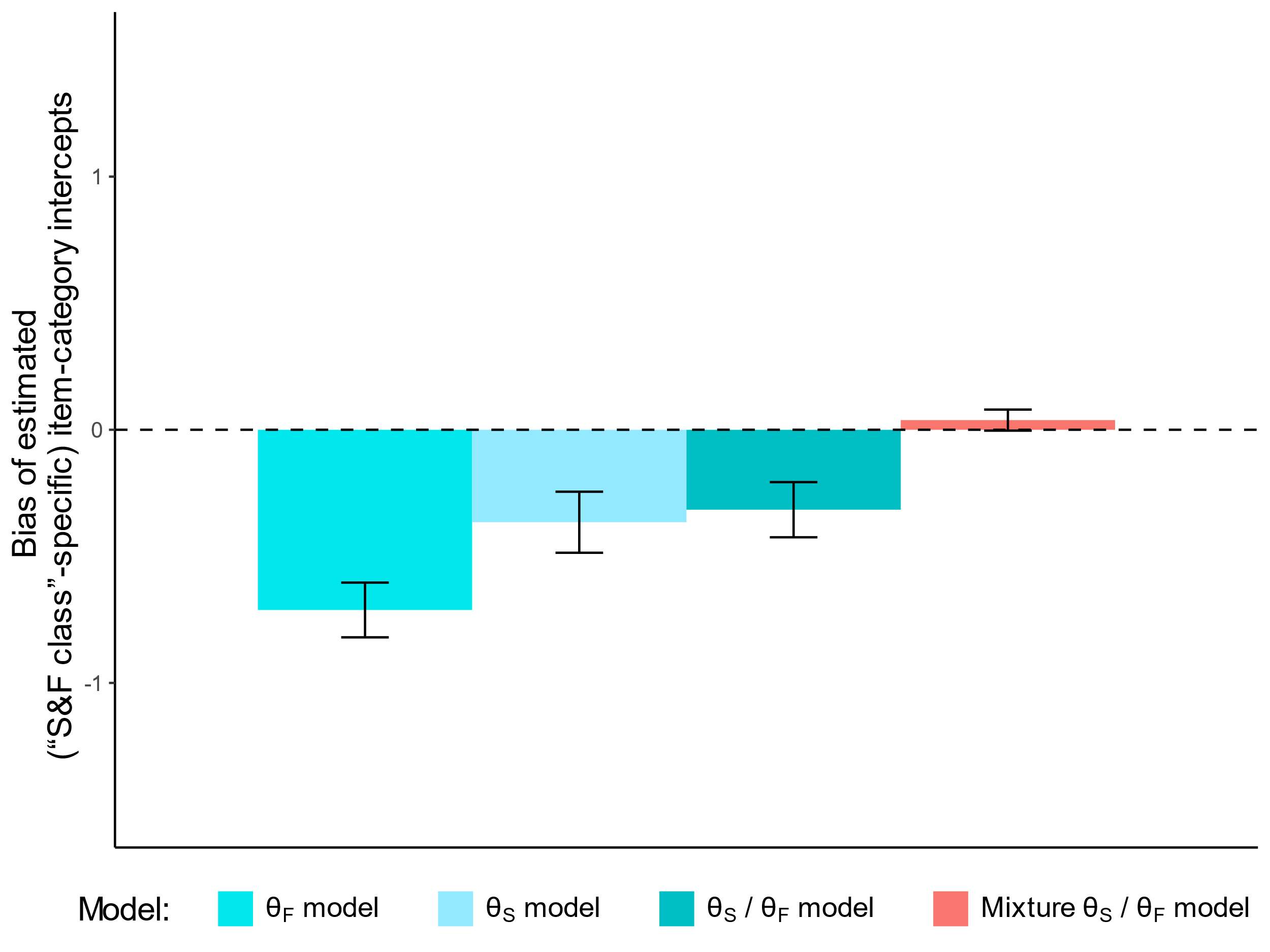

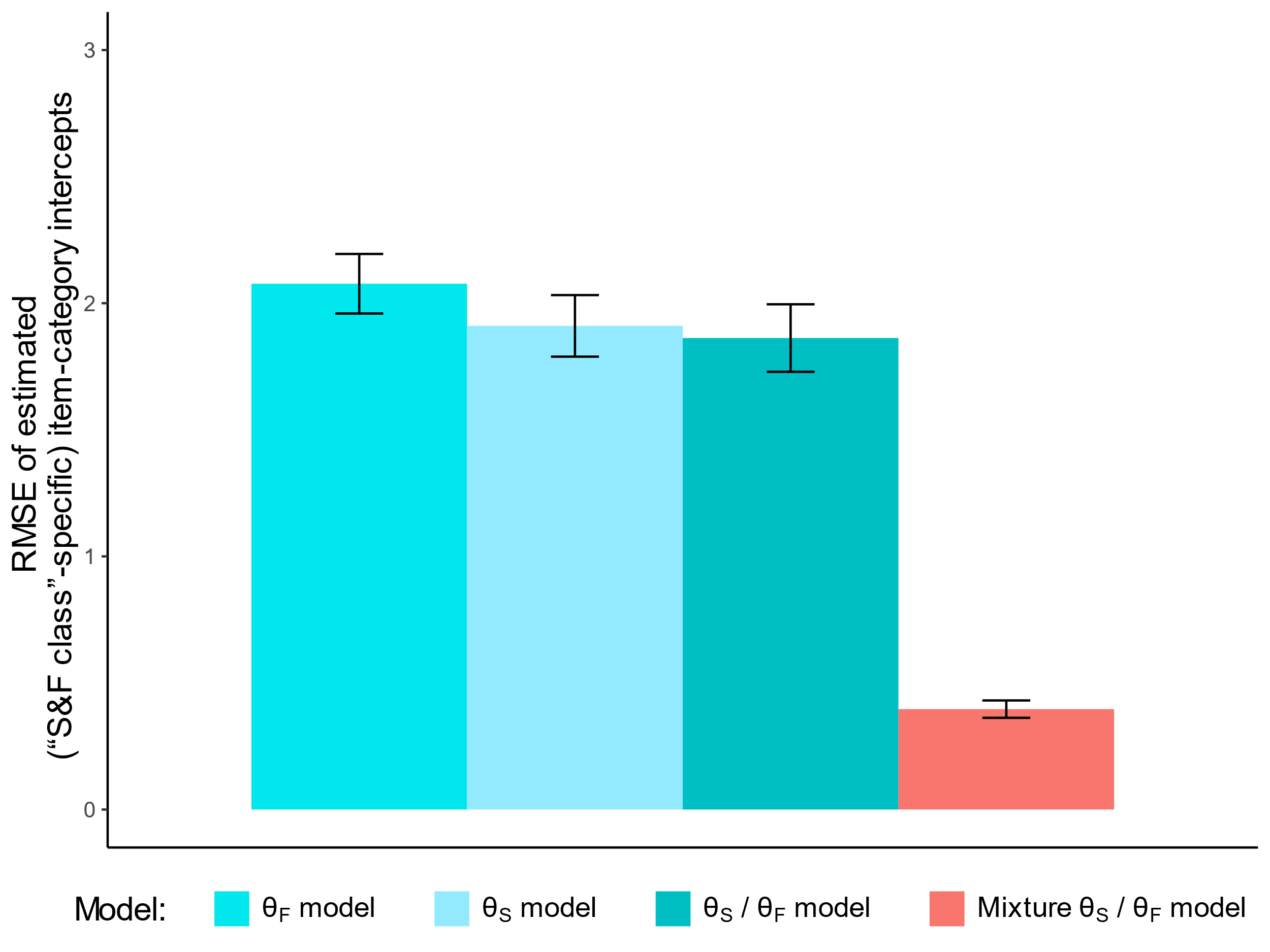


b) Recovery of Item-Category Intercepts of the “S-only Class”


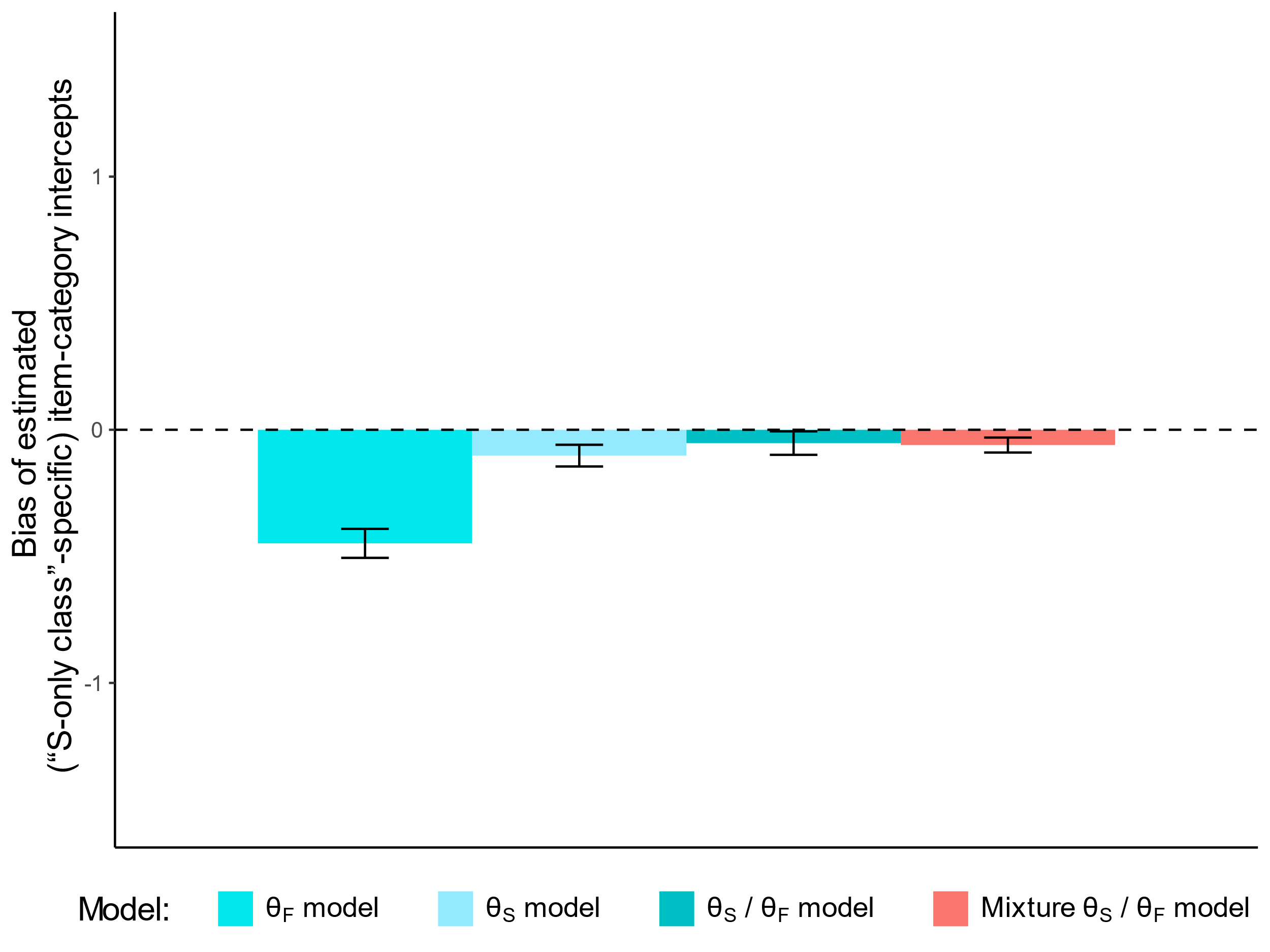

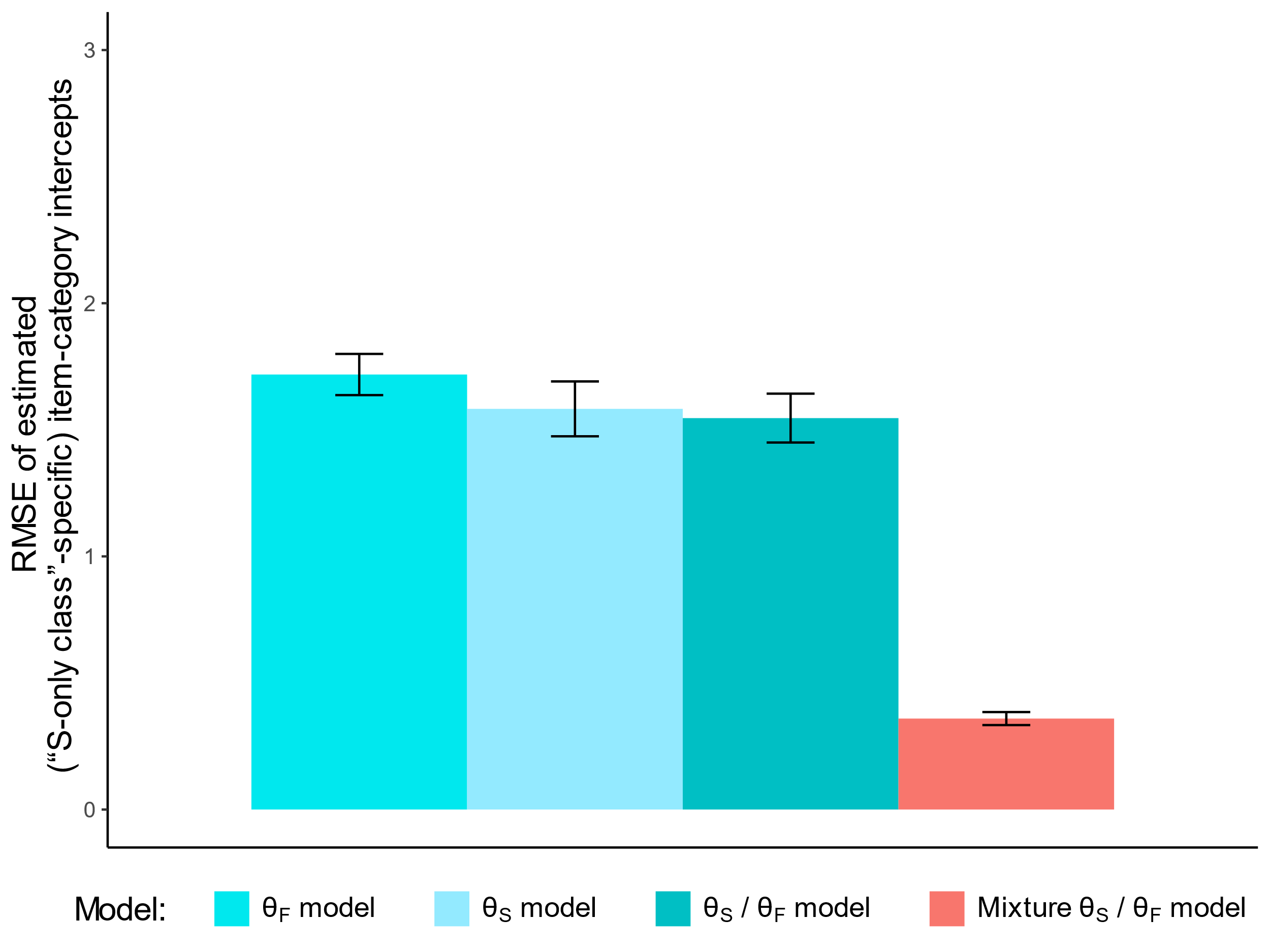


*(Continued on next page)*

b) Recovery of Item-Category Intercepts of the “F-only Class”


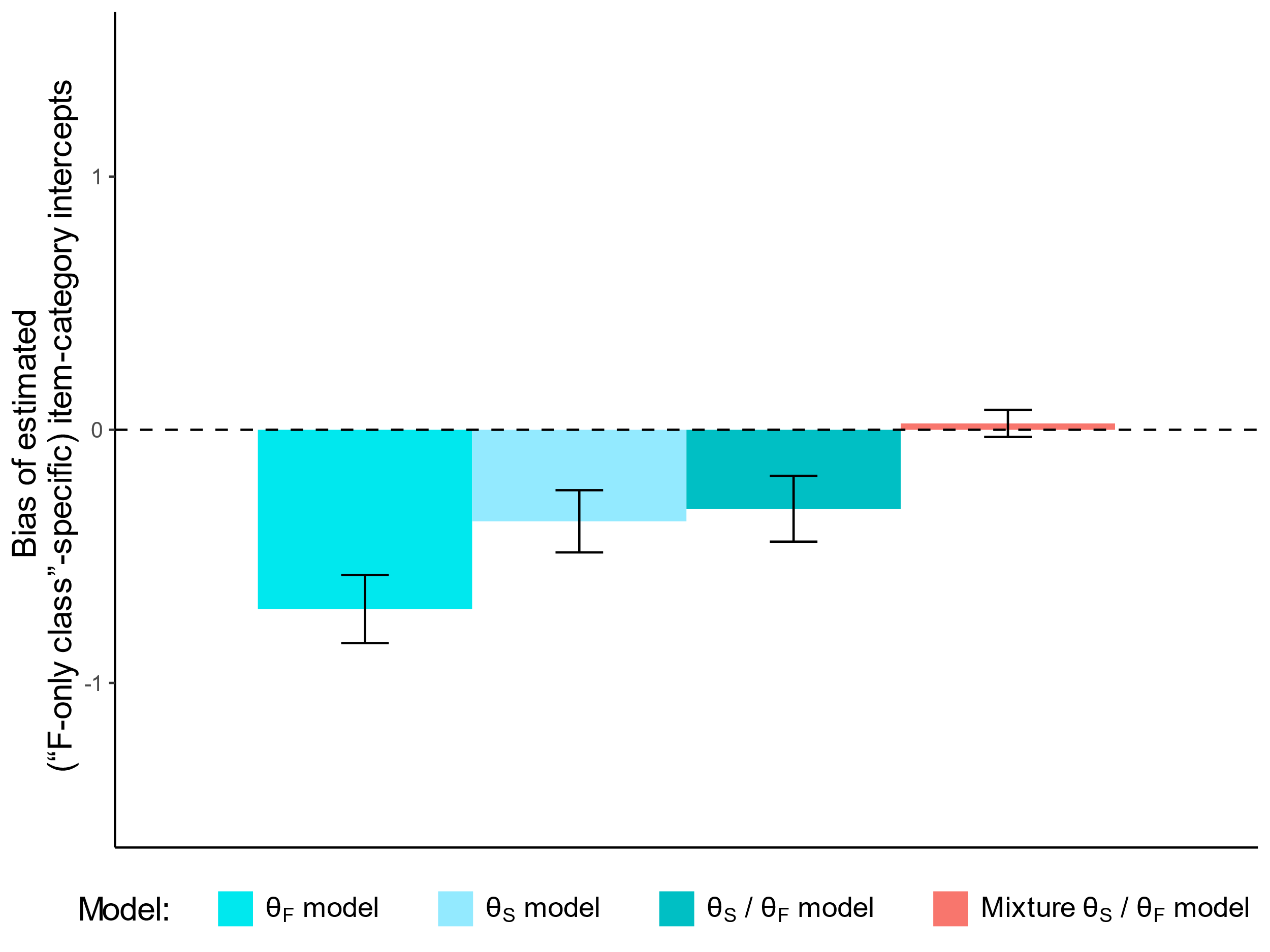

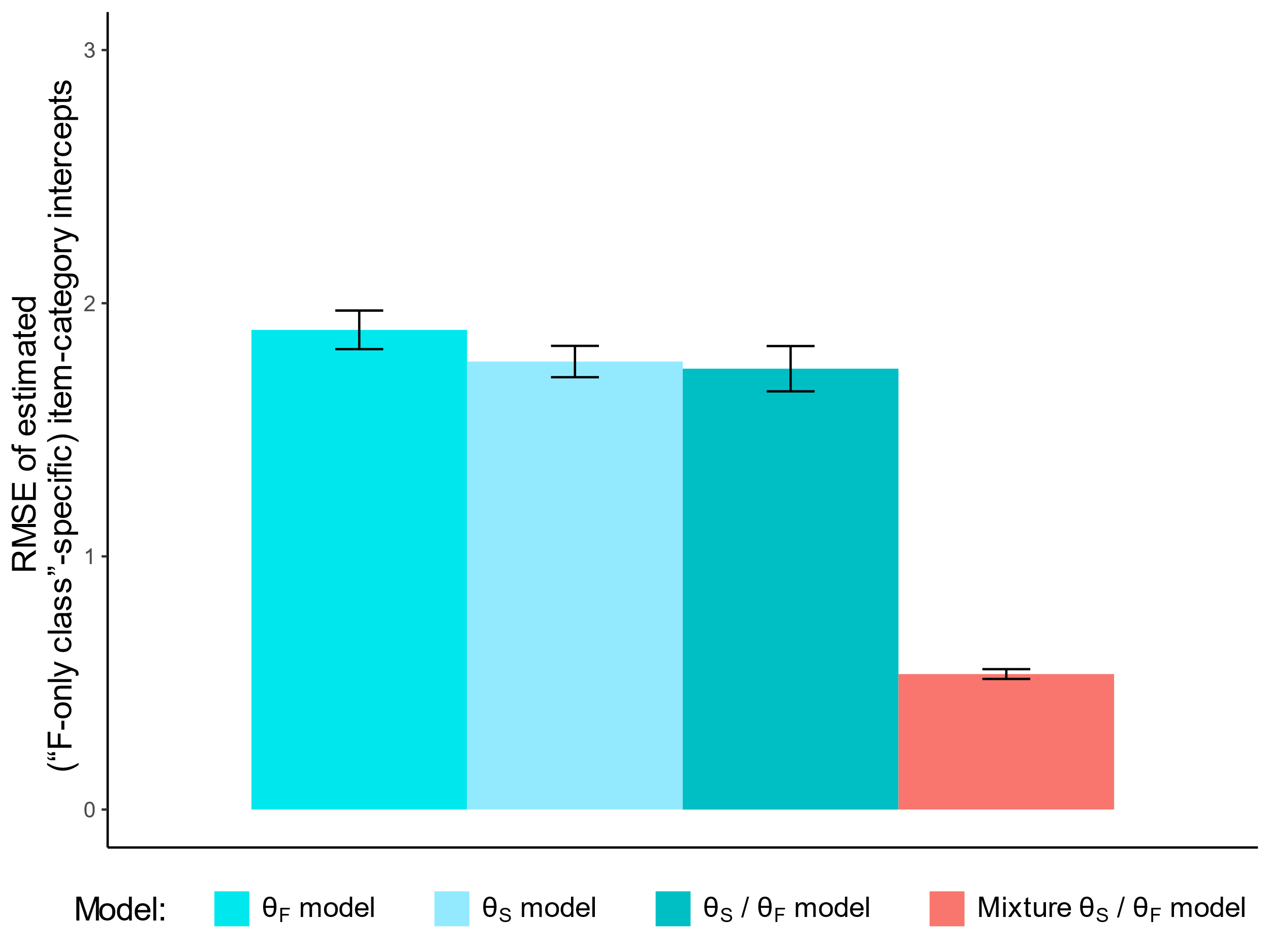


*Note.* Values reflect the mean bias or root mean square error (RMSE) of estimated item-category intercepts with respect to the true values in the “S&F class” (Panel a), “S-only class” (Panel b), or “F-only class” (Panel c) across replications. For the mixture model, the respective class-specific intercept estimates are considered. Error bars represent the standard error of the mean.

**Figure S10**

*Recovery of Latent Correlations in the Simulation With Non-Equidistant Scoring Weights of Faking*

a) Recovery of Latent Correlations Between Substantive Traits


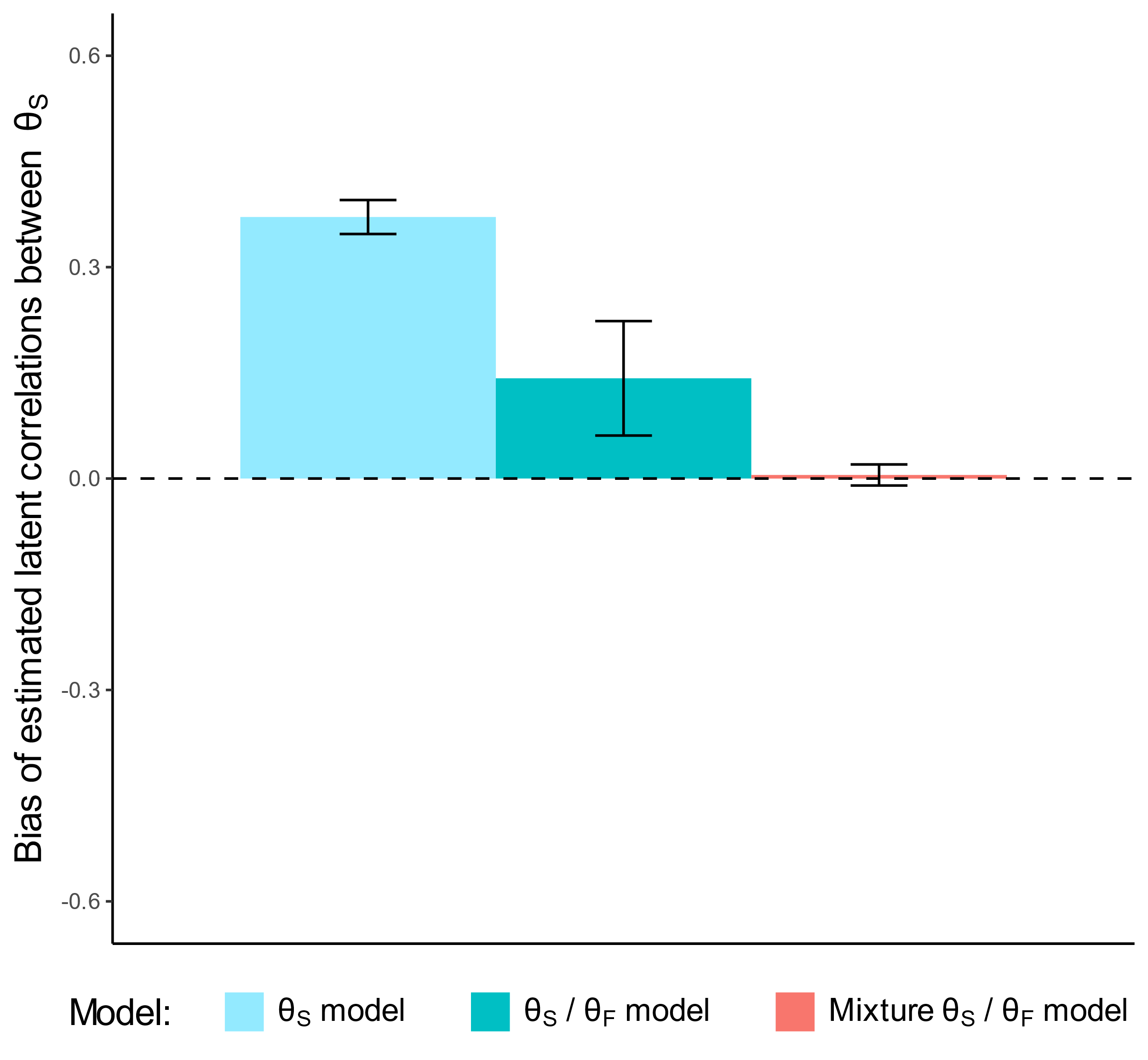

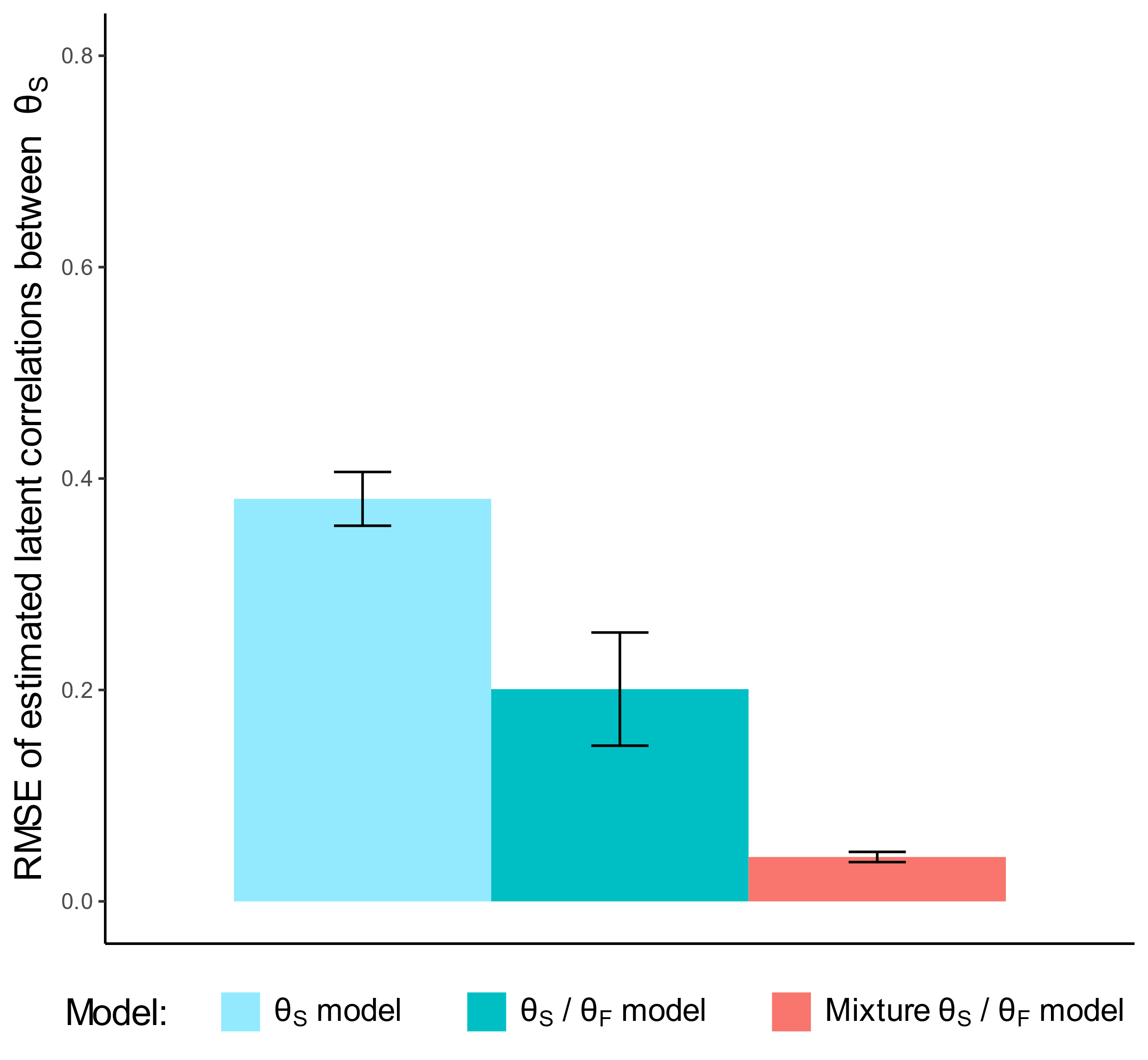


b) Recovery of Latent Correlations Between Faking and Substantive Traits


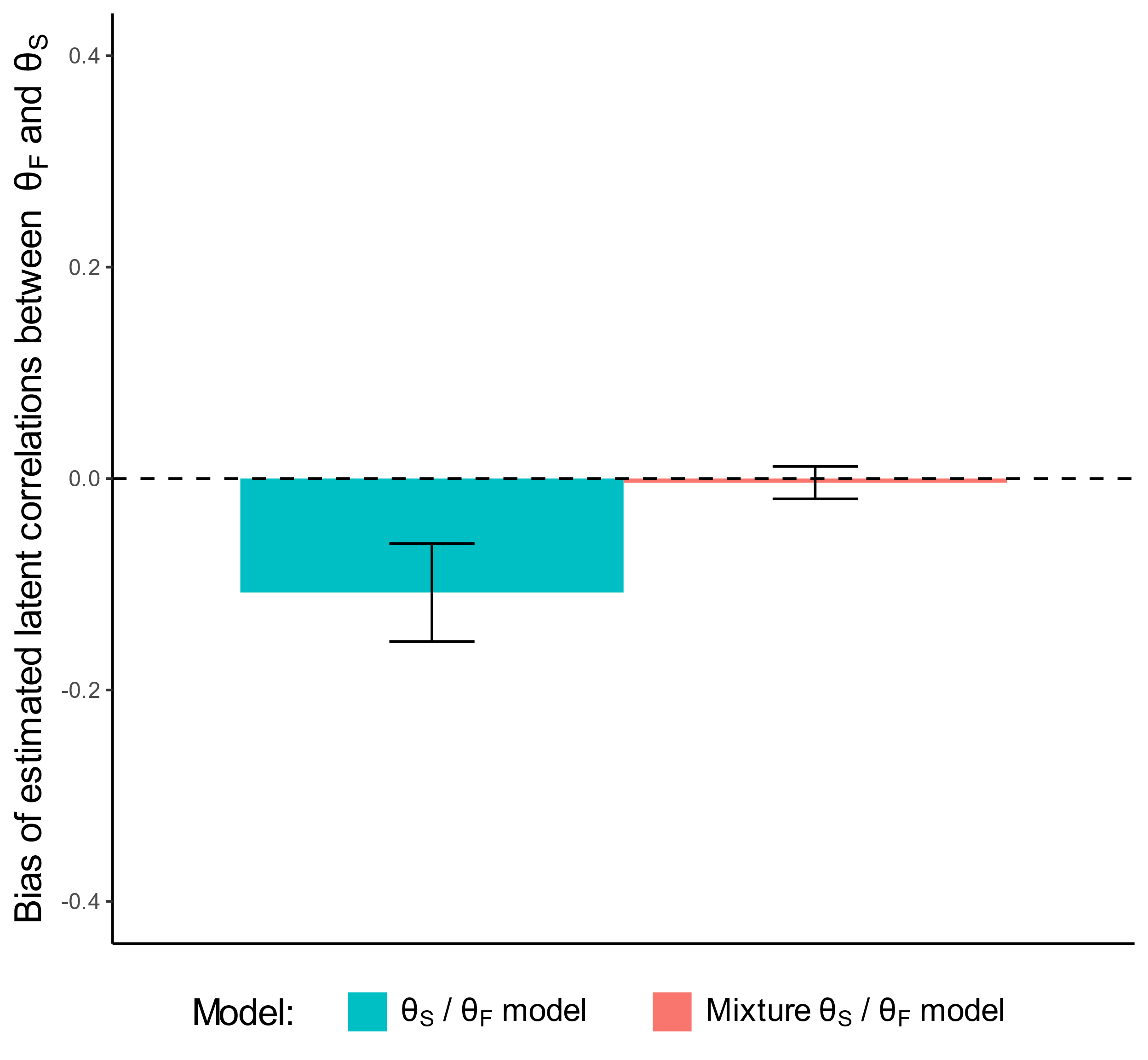

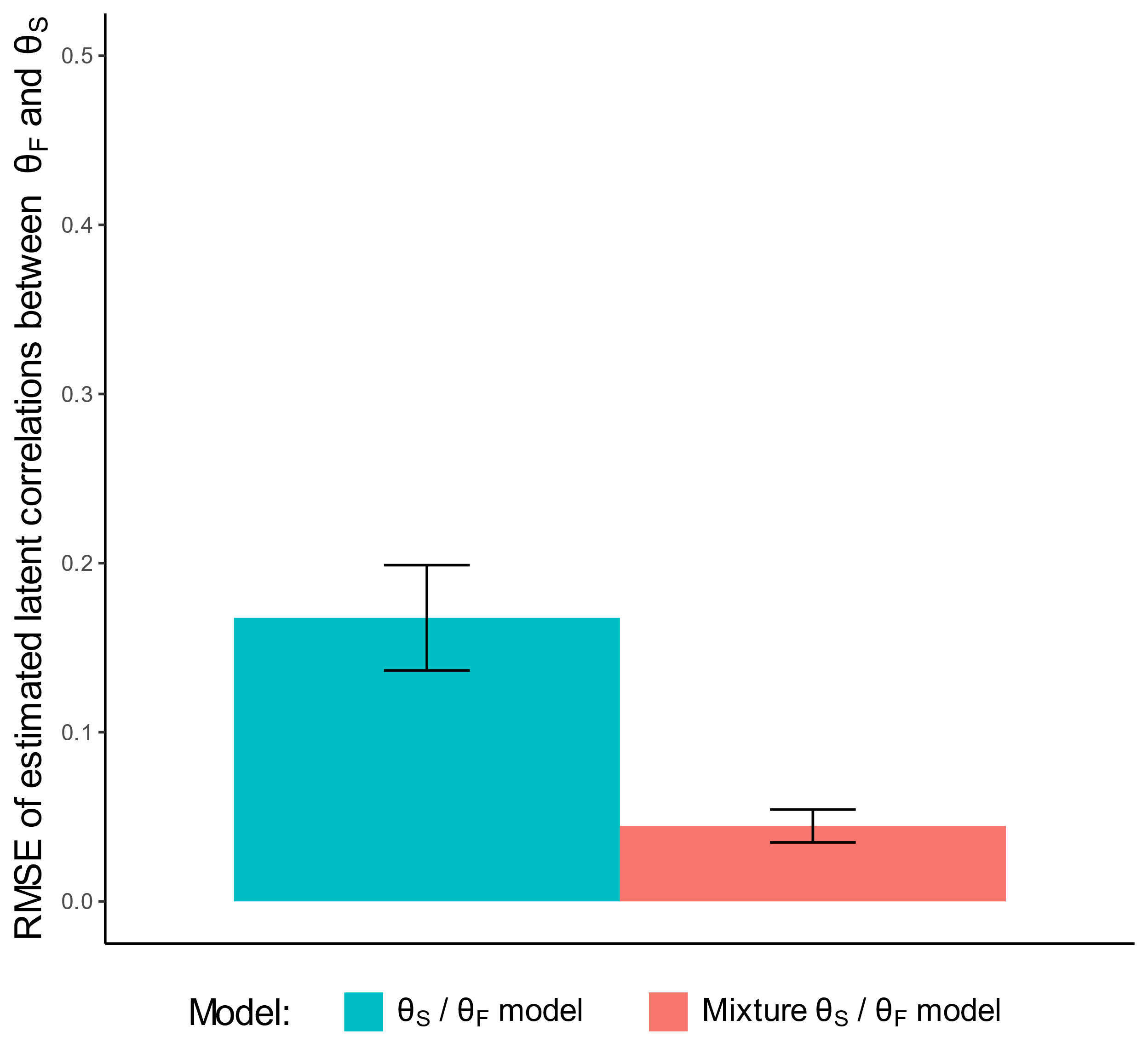


*Note.* Values reflect the mean bias or root mean square error (RMSE) of estimated latent correlations between substantive traits (Panel a) or faking and substantive traits (Panel b) across replications. Results are aggregated across the three substantive traits used in the simulation. Error bars represent the standard error of the mean.

## Details on the Pilot Studies

To determine scoring weights of faking for the empirical demonstration of the M-MNRM in the three datasets described in the Main Text, we ran a series of pilot studies in which participants should rate the social desirability of every response category for every item of the personality test used in the respective selection assessment. Because the datasets were from three different job application contexts (Dataset 1: application for a bank apprenticeship; Dataset 2: application for a police officer traineeship; Dataset 3: application for a position as insurance agent), we conducted separate pilot studies that were tailored to assess the items’ desirability characteristics with respect to the three personnel selection settings. The procedure, however, was constant across the three pilot studies (cf. Kuncel & Tellegen, 2009; Seitz et al., 2025). The pilot study for the assessment of desirability characteristics in the context of the bank apprenticeship application (Pilot Study 1) was already reported by Seitz et al. (2025).

### Procedure and Material

After participants completed demographic measures, we instructed them to take the perspective of a person currently applying for either an apprenticeship at a large German bank (Pilot Study 1), a traineeship as police officer at a German police department (Pilot Study 2), or a position as insurance agent at a German insurance company (Pilot Study 3). We then familiarized participants with typical tasks of the respective job before telling them that a questionnaire on personal attitudes and behaviors would be part of the application process. Next, we instructed them that, for every statement of the questionnaire (i.e., item), they should indicate how desirable they assess each of seven graded agreement levels (i.e., response categories) in the context of the respective job application. Ratings were given on a 5-point Kunin scale (Kunin, 1955; 0 = *not desirable* to 4 = *desirable*). As in the actual selection assessments, items were presented on separate questionnaire pages and in a random order. We used the online platform *SoSci Survey* (<https://www.soscisurvey.de/>) for data collection. Materials, data, and the analysis script of the pilot studies can be found at <https://osf.io/vwqf3/>.

### Samples

Since desirability ratings collected in the pilot studies should be valid approximations of desirability perceptions in the actual applicant populations, the participant samples in the pilot studies had to be maximally similar to the actual samples of applicants. Based on available demographic information on the applicant populations, we allowed participation in the respective pilot study only if,

- for the assessment of desirability characteristics in the context of the bank apprenticeship application (Pilot Study 1), participants were not older than 23 years, had completed at least 10 years of schooling, did not (yet) hold a university or vocational training degree, had graduated from school not more than 4 years ago, and had less than 12 months of full-time work experience,
- for the assessment of desirability characteristics in the context of the police officer traineeship application (Pilot Study 2), participants were not older than 30 years and had completed at least 10 years of schooling,
- and, for the assessment of desirability characteristics in the context of the application as insurance agent (Pilot Study 3), participants were aged between 18 and 60 and had completed at least 10 years of schooling.

Because each pilot study should accurately assess desirability values, the sample size rationale was to reach a predefined measurement precision. In particular, we sampled participants until, per pilot study, a) the median standard error of the mean of desirability ratings was smaller than 0.10 and b) the maximum standard error of the mean fell below 0.20. In Pilot Study 1, this resulted in a sample $N=63$ participants, with 63.5% being female (36.5% male) and a mean age of $M=20.63$ years ($SD=1.17$, $range=[18, 23]$). In Pilot Study 2, there were $N=74$ participants, of whom 32.4% were female (67.6% male) and the mean age was $M=21.93$ years ($SD=2.23$, $range=[18, 29]$). In Pilot Study 3, the sample consisted of $N=65$ participants, with 32.3% females (67.7% males) and a mean age of $M=23.88$ years ($SD=6.74$, $range=[18, 57]$). Most participants (Pilot Study 1: 77.7%; Pilot Study 2: 82.4% 2; Pilot Study 3: 76.9%) were undergraduate psychology students.

### Results

Mean desirability ratings were computed for each of the seven response categories for each item of the personality test used in the respective selection assessment. Results can be found in Table S5.^[[3]](#footnote-4)^ When reversing the order of categories for negatively-keyed items, 6 items (19.4%) in Pilot Study 1 had their highest mean desirability rating at category '6', 14 items (45.2%) at category '5', 8 items (25.8%) at category '4', 2 items (6.5%) at category '3', and 1 item (3.2%) at category '2'. In Pilot Study 2, 10 items (32.2%) had a highest-desirability category of '6', 11 items (35.5%) of '5', 7 items (22.6%) of '4', 2 items (6.5%) of '3', and 1 item (3.2%) of '0'. 10 items (32.3%) in Pilot Study 3 had a highest-desirability category of '6', 14 items (45.2%) of '5', 5 items (16.1%) of '4', 1 item (3.2%) of '2', and 1 item (3.2%)
of '0'.

**Table S8**

*Mean Desirability Ratings of Each Item in the Three Pilot Studies*

|  | Response category | | | | | | | | | | | | | | | | | | | | | | |
| --- | --- | --- | --- | --- | --- | --- | --- | --- | --- | --- | --- | --- | --- | --- | --- | --- | --- | --- | --- | --- | --- | --- | --- |
| Item no. | 0 | 1 | 2 | 3 | 4 | 5 | 6 |  | 0 | 1 | 2 | 3 | 4 | 5 | 6 |  | 0 | 1 | 2 | 3 | 4 | 5 | 6 |
|  | *Pilot Study 1 (bank apprenticeship)* | | | | | | |  | *Pilot Study 2 (police officer traineeship)* | | | | | | |  | *Pilot Study 3 (position as insurance agent)* | | | | | | |
| 1 (R) | 4.83 | 4.25 | 3.44 | 2.27 | 1.56 | 1.22 | 1.11 |  | 4.70 | 4.22 | 3.55 | 2.39 | 1.74 | 1.36 | 1.26 |  | 4.75 | 4.32 | 3.57 | 2.46 | 1.68 | 1.25 | 1.15 |
| 2 (R) | 4.92 | 4.37 | 3.54 | 2.13 | 1.51 | 1.17 | 1.11 |  | 4.66 | 4.22 | 3.49 | 2.26 | 1.64 | 1.27 | 1.18 |  | 4.02 | 3.77 | 3.52 | 2.89 | 2.42 | 2.15 | 1.91 |
| 3 (R) | 4.29 | 4.22 | 3.70 | 2.65 | 1.97 | 1.67 | 1.49 |  | 4.31 | 3.91 | 3.50 | 2.68 | 2.09 | 1.76 | 1.53 |  | 4.29 | 3.97 | 3.55 | 2.54 | 1.88 | 1.38 | 1.28 |
| 4 (R) | 3.40 | 3.86 | 3.89 | 3.22 | 2.62 | 2.00 | 1.79 |  | 4.05 | 4.03 | 3.68 | 2.89 | 2.07 | 1.64 | 1.43 |  | 3.52 | 3.66 | 3.63 | 3.26 | 2.72 | 2.34 | 2.02 |
| 5 (R) | 4.73 | 4.33 | 3.62 | 2.48 | 1.86 | 1.35 | 1.16 |  | 4.47 | 4.19 | 3.82 | 2.81 | 2.09 | 1.54 | 1.31 |  | 4.68 | 4.11 | 3.51 | 2.54 | 1.72 | 1.29 | 1.15 |
| 6 | 2.10 | 2.54 | 3.17 | 3.56 | 3.63 | 3.22 | 2.84 |  | 2.24 | 2.70 | 3.05 | 3.47 | 3.59 | 3.49 | 3.23 |  | 2.42 | 2.63 | 2.89 | 3.35 | 3.58 | 3.55 | 3.28 |
| 7 (R) | 2.79 | 3.19 | 3.44 | 3.40 | 3.27 | 2.86 | 2.57 |  | 3.15 | 3.38 | 3.47 | 3.28 | 2.97 | 2.58 | 2.34 |  | 3.95 | 3.83 | 3.60 | 2.98 | 2.46 | 1.98 | 1.83 |
| 8 (R) | 1.89 | 2.40 | 3.11 | 3.86 | 3.95 | 3.51 | 3.14 |  | 2.99 | 3.41 | 3.57 | 3.28 | 2.96 | 2.54 | 2.16 |  | 2.08 | 2.52 | 3.03 | 3.55 | 3.62 | 3.32 | 3.02 |
| 9 | 1.16 | 1.48 | 2.11 | 3.24 | 4.35 | 4.76 | 4.63 |  | 1.14 | 1.47 | 2.15 | 3.14 | 4.15 | 4.77 | 4.80 |  | 1.20 | 1.51 | 2.08 | 3.22 | 4.34 | 4.83 | 4.75 |
| 10 | 1.19 | 1.44 | 2.11 | 3.19 | 4.30 | 4.84 | 4.76 |  | 1.16 | 1.58 | 2.22 | 3.24 | 4.30 | 4.78 | 4.84 |  | 1.20 | 1.66 | 2.20 | 3.29 | 4.28 | 4.85 | 4.89 |
| 11 (R) | 3.19 | 3.65 | 3.84 | 3.51 | 2.67 | 2.03 | 1.70 |  | 3.96 | 3.95 | 3.74 | 3.04 | 2.36 | 1.68 | 1.54 |  | 3.71 | 3.86 | 3.68 | 3.20 | 2.49 | 1.98 | 1.75 |
| 12 | 2.08 | 2.49 | 3.02 | 3.73 | 3.76 | 3.38 | 3.10 |  | 1.84 | 2.20 | 2.70 | 3.42 | 3.95 | 3.89 | 3.76 |  | 1.94 | 2.38 | 2.89 | 3.48 | 3.83 | 3.68 | 3.45 |
| 13 | 1.21 | 1.41 | 1.94 | 2.98 | 3.97 | 4.70 | 4.78 |  | 3.61 | 3.51 | 3.34 | 2.88 | 2.46 | 2.19 | 2.12 |  | 1.22 | 1.49 | 1.97 | 2.92 | 4.18 | 4.74 | 4.80 |
| 14 | 2.25 | 2.76 | 3.27 | 3.89 | 3.65 | 3.06 | 2.63 |  | 2.77 | 3.04 | 3.47 | 3.61 | 3.26 | 2.93 | 2.59 |  | 1.74 | 2.12 | 2.78 | 3.49 | 4.09 | 4.20 | 3.88 |
| 15 (R) | 3.11 | 3.46 | 3.65 | 3.60 | 2.95 | 2.37 | 2.08 |  | 3.99 | 3.97 | 3.76 | 2.89 | 2.27 | 1.69 | 1.53 |  | 3.22 | 3.29 | 3.46 | 3.29 | 3.00 | 2.68 | 2.43 |
| 16 | 1.29 | 1.68 | 2.19 | 3.33 | 4.29 | 4.65 | 4.43 |  | 1.18 | 1.45 | 2.07 | 3.24 | 4.36 | 4.88 | 4.80 |  | 1.71 | 2.12 | 2.71 | 3.45 | 4.05 | 4.14 | 4.00 |
| 17 | 1.17 | 1.49 | 2.03 | 3.08 | 4.21 | 4.87 | 4.81 |  | 1.49 | 1.95 | 2.49 | 3.47 | 4.30 | 4.55 | 4.49 |  | 1.26 | 1.62 | 2.22 | 3.25 | 4.34 | 4.80 | 4.68 |
| 18 | 1.22 | 1.60 | 2.24 | 3.29 | 4.24 | 4.70 | 4.59 |  | 1.47 | 1.96 | 2.65 | 3.55 | 4.16 | 4.34 | 4.23 |  | 1.29 | 1.63 | 2.12 | 3.20 | 4.22 | 4.58 | 4.45 |
| 19 (R) | 3.27 | 3.71 | 3.90 | 3.67 | 2.86 | 2.33 | 2.08 |  | 3.51 | 3.73 | 3.69 | 3.27 | 2.74 | 2.32 | 2.08 |  | 3.88 | 3.89 | 3.63 | 3.02 | 2.40 | 1.94 | 1.75 |
| 20 (R) | 2.60 | 3.14 | 3.67 | 3.75 | 3.33 | 2.94 | 2.68 |  | 2.41 | 2.85 | 3.42 | 3.72 | 3.54 | 3.26 | 3.03 |  | 3.11 | 3.32 | 3.49 | 3.42 | 2.92 | 2.55 | 2.32 |
| 21 | 1.03 | 1.37 | 2.10 | 3.21 | 4.38 | 4.83 | 4.79 |  | 1.64 | 2.22 | 2.81 | 3.69 | 4.08 | 4.05 | 3.72 |  | 1.14 | 1.46 | 2.03 | 3.02 | 4.34 | 4.86 | 4.85 |
| 22 | 1.06 | 1.43 | 2.14 | 3.10 | 4.29 | 4.76 | 4.32 |  | 1.31 | 1.84 | 2.43 | 3.28 | 4.26 | 4.58 | 4.28 |  | 1.20 | 1.46 | 1.92 | 2.98 | 4.09 | 4.77 | 4.86 |
| 23 | 1.13 | 1.52 | 2.19 | 3.32 | 4.32 | 4.65 | 4.27 |  | 1.50 | 2.00 | 2.62 | 3.57 | 4.19 | 4.23 | 3.95 |  | 1.42 | 1.80 | 2.37 | 3.37 | 4.35 | 4.45 | 4.14 |
| 24 | 1.57 | 1.90 | 2.52 | 3.38 | 4.08 | 4.25 | 3.97 |  | 1.66 | 2.19 | 2.66 | 3.50 | 4.23 | 4.16 | 3.97 |  | 3.66 | 3.60 | 3.52 | 2.89 | 2.35 | 1.86 | 1.66 |
| 25 | 1.29 | 1.56 | 2.21 | 3.19 | 4.10 | 4.71 | 4.57 |  | 1.57 | 2.00 | 2.58 | 3.45 | 4.22 | 4.49 | 4.38 |  | 1.34 | 1.86 | 2.49 | 3.42 | 4.34 | 4.66 | 4.55 |
| 26 | 1.52 | 1.89 | 2.59 | 3.83 | 4.51 | 4.11 | 3.30 |  | 2.09 | 2.46 | 2.99 | 3.68 | 3.80 | 3.38 | 2.93 |  | 1.77 | 2.22 | 2.95 | 3.95 | 4.08 | 3.69 | 3.23 |
| 27 | 1.06 | 1.29 | 1.84 | 2.97 | 4.27 | 4.94 | 4.89 |  | 1.18 | 1.58 | 2.14 | 3.22 | 4.27 | 4.88 | 4.88 |  | 1.18 | 1.48 | 1.89 | 3.05 | 4.31 | 4.83 | 4.83 |
| 28 | 1.05 | 1.21 | 1.83 | 3.00 | 4.29 | 4.87 | 4.65 |  | 1.12 | 1.42 | 2.07 | 3.15 | 4.22 | 4.73 | 4.59 |  | 1.20 | 1.46 | 2.09 | 2.97 | 4.26 | 4.86 | 4.77 |
| 29 | 1.19 | 1.51 | 2.16 | 3.32 | 4.32 | 4.73 | 4.40 |  | 1.51 | 1.97 | 2.43 | 3.49 | 4.26 | 4.54 | 4.27 |  | 1.43 | 1.92 | 2.52 | 3.31 | 4.35 | 4.69 | 4.45 |
| 30 | 1.05 | 1.32 | 1.98 | 3.00 | 4.17 | 4.83 | 4.89 |  | 1.08 | 1.54 | 2.15 | 3.14 | 4.31 | 4.80 | 4.88 |  | 1.23 | 1.54 | 2.06 | 3.12 | 4.29 | 4.82 | 4.88 |
| 31 | 1.24 | 1.68 | 2.19 | 3.32 | 4.33 | 4.67 | 4.16 |  | 1.41 | 1.86 | 2.47 | 3.46 | 4.18 | 4.20 | 3.95 |  | 1.37 | 1.69 | 2.32 | 3.40 | 4.43 | 4.57 | 4.11 |
|  |  |  |  |  |  |  |  |  |  |  |  |  |  |  |  |  |  |  |  |  |  |  |  |

*Note.* Mean desirability ratings are based on $N=63$ (Pilot Study 1), $N=74$ (Pilot Study 2), and $N=65$ (Pilot Study 3) participants. Ratings had a possible range from 1 to 5. For model estimation, however, mean ratings were linearly transformed to a possible range from 0 to 6 before being used as scoring weights of the faking dimension. Also, the order of faking scoring weights for negatively-keyed items (R) had to be reversed because responses to these items were recoded.

# References

Alagöz, Ö. E. C., & Meiser, T. (2024). Investigating heterogeneity in response strategies: A mixture multidimensional IRTree approach. *Educational and Psychological Measurement, 84*(5), 957–993. <https://doi.org/10.1177/00131644231206765>

Birkeland, S. A., Manson, T. M., Kisamore, J. L., Brannick, M. T., & Smith, M. A. (2006). A meta-analytic investigation of job applicant faking on personality measures. *International Journal of Selection and Assessment, 14*(4), 317–335. <https://doi.org/10.1111/j.1468-2389.2006.00354.x>

Hu, J., & Connelly, B. S. (2021). Faking by actual applicants on personality tests: A meta‐analysis of within‐subjects studies. *International Journal of Selection and Assessment, 29*(3-4), 412–426. <https://doi.org/10.1111/ijsa.12338>

Kuncel, N. R., & Tellegen, A. (2009). A conceptual and empirical reexamination of the measurement of the social desirability of items: Implications for detecting desirable response style and scale development. *Personnel Psychology, 62*(2), 201–228. <https://doi.org/10.1111/j.1744-6570.2009.01136.x>

Kunin, T. (1955). The construction of a new type of attitude measure. *Personnel Psychology, 8*(1), 65–77. <https://doi.org/10.1111/j.1744-6570.1955.tb01189.x>

Seitz, T., Spengler, M., & Meiser, T. (2025). “What if applicants fake their responses?”: Modeling faking and response styles in high-stakes assessments using the multidimensional nominal response model. *Educational and Psychological Measurement*. Advance online publication. <https://doi.org/10.1177/00131644241307560>

Seitz, T., Wetzel, E., Hilbig, B. E., & Meiser, T. (2024). Using the multidimensional nominal response model to model faking in questionnaire data: The importance of item desirability characteristics. *Behavior Research Methods, 56*(8), 8869–8896. <https://doi.org/10.3758/s13428-024-02509-x>

Viswesvaran, C., & Ones, D. S. (1999). Meta-analyses of fakability estimates: Implications for personality measurement. *Educational and Psychological Measurement, 59*(2), 197–210. <https://doi.org/10.1177/00131649921969802>

1. Note that, in the Bayesian model estimation we employed, person parameters are not derived after item and other model parameters are estimated, which is usually the case in frequentist estimation procedures (such as maximum likelihood). Person parameters are instead estimated together with all remaining parameters using MCMC sampling. Estimated substantive trait and faking scores thus represent estimates based on class-specific estimates weighted by the posterior class probabilities of the person, instead of representing estimates conditional on the person’s modal class. However, in case of high class separation, the two concepts do not make much of a difference. [↑](#footnote-ref-2)
2. The logic for the class-specific estimation and practical handling of faking scores is analogous. [↑](#footnote-ref-3)
3. Note that item wordings of the personality tests used in the three selection assessments were similar but not fully identical. This was because item wordings used in the applications for the bank apprenticeship and police officer traineeship had been adjusted to better fit the age group of applicants for the respective job (mainly high school graduates). [↑](#footnote-ref-4)
